# Father's influence on breastfeeding continuity or interruption: meta-synthesis

**DOI:** 10.1590/1980-220X-REEUSP-2024-0303en

**Published:** 2025-04-11

**Authors:** Willyane de Andrade Alvarenga, Thais Rayane da Conceição Gomes, Carlos Henrique Rodrigues da Silva, Giovanna Cristina Machado Kayzuka, Francine de Montigny, Adriana Moraes Leite, Lucila Castanheira Nascimento

**Affiliations:** 1 Centro Universitário Santo Agostinho, Teresina, PI, Brazil.; 2 Universidade de São Paulo, Escola de Enfermagem de Ribeirão Preto, Ribeirão Preto, SP, Brazil.; 3 Université du Québec en Outaouais, Département des sciences infirmières, Gatineau, Canada.

**Keywords:** Fathers, Weaning, Breast Feeding, Qualitative Research

## Abstract

**Objective:**

To synthesize qualitative evidence on the father’s influence on the mother’s decision to maintain or interrupt breastfeeding.

**Method:**

Qualitative metasynthesis, with systematic searches in the LILACS, PubMed, Scopus, and CINAHL databases, supplemented by manual searches. ENTREQ (*Enhancing Transparency in Reporting the Synthesis of Qualitative Research*) recommendations and the checklist CASP (*Critical Appraisal Skills Program*) were followed to assess the studies quality. In the data synthesis process, a thematic synthesis approach was used.

**Results:**

Of a total of 1,158 references identified, 13 were included. The thematic synthesis allowed the construction of two analytical themes, which qualify paternal influence: ‘Difficulty in actively participating in the breastfeeding process’, with four descriptive themes, and ‘Acting directly or indirectly with the mother during breastfeeding’, with three descriptive themes.

**Conclusion:**

Most studies revealed the decision-making process to be centered on the mother. Health education aimed at fathers during prenatal care contributes to participatory fatherhood and encourages breastfeeding.

## INTRODUCTION

Breast milk is a complete food, of utmost importance for the baby’s nutritional, neurological, and immunological development^(1)^. It is capable of reducing the incidence of infant mortality due to preventable diseases, by helping to strengthen the immune system^(2)^. Children who are exclusively breastfed until six months of age have a lower incidence of hospitalization, mainly due to diarrhea^(3)^. Therefore, the World Health Organization (WHO) recommends maintaining exclusive on-demand breastfeeding until the baby is six months old^(4)^. After this period, it is recommended to introduce complementary feeding, but maintaining breastfeeding in a complementary manner until two years or more^(5)^.

Despite the benefits of breastfeeding for infants, the number of women who exclusively breastfeed their children remains low, as many opt to use supplements, such as infant formulas^(6)^. As a result of early weaning, children may develop health problems such as acute diarrhea, anemia, nutritional deficiencies, greater susceptibility to acquiring chronic diseases such as diabetes, infections and respiratory diseases such as bronchitis and asthma, in addition to an increased risk of death due to lack of nutrients^(7)^. Among the factors that contribute to early weaning are difficulties related to the lack of guidance on breastfeeding, the need to return to work before the baby is six months old, psychological suffering, and the absence of a support network to maintain breastfeeding^(8,9,10,11)^.

The availability of a support network, made up of family, friends, or health professionals, is essential for strengthening women’s physical, social, psychological, cultural, and professional aspects^(12)^. In general, such support is represented by other women in the social circle, such as mother, mother-in-law, or aunts, especially in the first months after childbirth^(12)^. Contact with these women’s history and experiences of breastfeeding, in addition to providing tools to overcome frequent difficulties during this period, interferes in the maintenance of breastfeeding by these mothers^(12,13)^.

The presence of the partner as a support network, however, is not clear in the literature. While some evidence highlights the low paternal willingness to participate in breastfeeding^(12)^, others show that these represent the largest support network for postpartum women^(13)^. The lack of paternal support harms the quality of breastfeeding, especially due to the accumulation of household tasks for these mothers, which leads to feelings of loneliness, withdrawal from medical care, and the belief that milk is insufficient to nourish their children^(12,13)^. This way, partner support is recognized as a turning point in the decision to continue breastfeeding, in addition to helping to create a family model focused on well-being and on strengthening bonds^(13,14)^.

Thus, the lack of paternal support and the resulting family problems are factors that interfere with successful breastfeeding^(15)^. Recognition of the father and his importance during pregnancy contributes to the child’s healthy growth, breastfeeding, and the partner’s postpartum period^(13,16)^. The father, in fact, shows himself to be the main agent in finding ways to help and comfort his wife/partner^(17)^, mainly during breastfeeding^(17,18)^. However, it is still unclear in the literature how it influences the mother’s decision to continue or stop breastfeeding her child^(19)^, nor are there literature reviews of qualitative studies based on the paternal perspective.

Given the above and the importance of further investigating this topic, this research aimed to synthesize qualitative evidence on the father’s influence on the mother’s decision to maintain or interrupt breastfeeding. To this end, the following research question was defined: “What is the qualitative evidence about paternal experience in deciding whether to continue or interrupt breastfeeding?”

## METHOD

### Design Of Study

This is a qualitative metasynthesis, which consists of a broad analysis of studies with a qualitative approach, contributing to the understanding of a particular problem^(20)^. For data synthesis, the approach used was that proposed by Thomas and Harden^(21)^ of the thematic synthesis of qualitative studies, according to the following steps: search for relevant studies; assessment of the quality of qualitative studies; data extraction; and thematic synthesis. Recommendations described in ENTREQ (*Enhancing Transparency in Reporting the Synthesis of Qualitative Research*) were used to report this qualitative metasynthesis^(22)^.

### Population, Local and Selection Criteria

The search was carried out by two reviewers, independently, to survey primary studies in the PubMed, Scopus, CINAHL and LILACS databases. A manual search was also carried out in the reference lists of the included articles. Studies in Portuguese, Spanish, and English were selected based on the fluency of the responsible research group, with the publication period from January 2012 to December 2023. The selected period ensures that the review includes up-to-date data, aligned with current practices and health policies to encourage paternal participation in breastfeeding, which were reflected in the introduction of the Child-Friendly Hospital Initiative, initiated by WHO and UNICEF^(4)^. This time frame allows for a focused review of research that reflects the evolution of these guidelines and approaches.

The different descriptors and keywords related to ‘pai/*father*’, ‘desmame/*Weaning*’ and ‘amamentação/*Breast Feeding*’ were selected according to the Health Sciences Descriptors (DeCS) and the *Medical Subject Headings* (MeSH) and combined using Boolean operators (AND, OR). The SPIDER tool (sample, phenomenon of interest, design, evaluation and type of research) was used to structure the search strategy to be used in the Pubmed database (Chart 1) and adapted to other databases, according to the specificities of each one^(23)^.

**Chart 1:** Search terms used in the Pubmed database – Teresina, PI, Brazil, 2024.

SPIDER	DESCRIPTION	SEARCH TERMS (DeCs, MeSH and keywords)
*Sample* (Amostra)	Father	“Fathers”[Mesh] OR “Fathers” OR ”Father” OR “Spouses”[Mesh] OR “Spouses” OR “Spouse”
*Phenomenon of Interest*(Fenômeno de interesse)	Breastfeeding	(“Breast Feeding”[Mesh] OR (Breastfed) OR (Breastfeeding)
*Design* (Desenho)	Qualitative studies Methods and techniques	“Focus Groups”[Mesh] OR (Focus Group) OR (Group, Focus) OR “Anthropology, Medical”[Mesh] OR (Medical Anthropology) OR “Grounded Theory”[Mesh] OR (Theory, Grounded) OR Culture OR (Thematic synthesis) OR “Hermeneutics”[Mesh] OR (Hermeneutic) OR (Ethnographic) OR (ethnographic research) OR (Phenomenology) OR (phenomenological research) OR (Narrative) OR “Interviews as Topic”[Mesh] OR (Interviewers) OR (Interviewer) OR (Interviewees) OR (Group Interviews) OR (Group Interview) OR (Interview, Group) OR (Interviews, Group) OR (in-depth interview) OR (qualitative interview) OR (content analysis) OR (semantic analysis)
*Evaluation* (Resultado)	Early weaning	“Weaning”[Mesh] OR (weaning)
*Research Type*(Tipo de pesquisa)	Qualitative research	“Qualitative Research”[Mesh] OR (Research, Qualitative) OR (Qualitative studies) OR (Qualitative) OR “Empirical Research”[Mesh] OR (Research, Empirical)

Source: Own authorship.

Primary studies with a qualitative approach on paternal experience during the breastfeeding period were included. Articles should have the father/man as a participant and studies with mixed samples would be included if the results relating to the father were presented and analyzed separately from the other participants. Mixed methods studies, in which qualitative results were presented and analyzed separately from quantitative data, would also be included. Literature review studies, quantitative research, editorials, conference abstracts, theses and dissertations, and studies with participants under 18 years of age or that jointly presented the experience of the father, mother, or health professional, indiscriminately, were excluded.

### Data Collection

**Figure 1:**
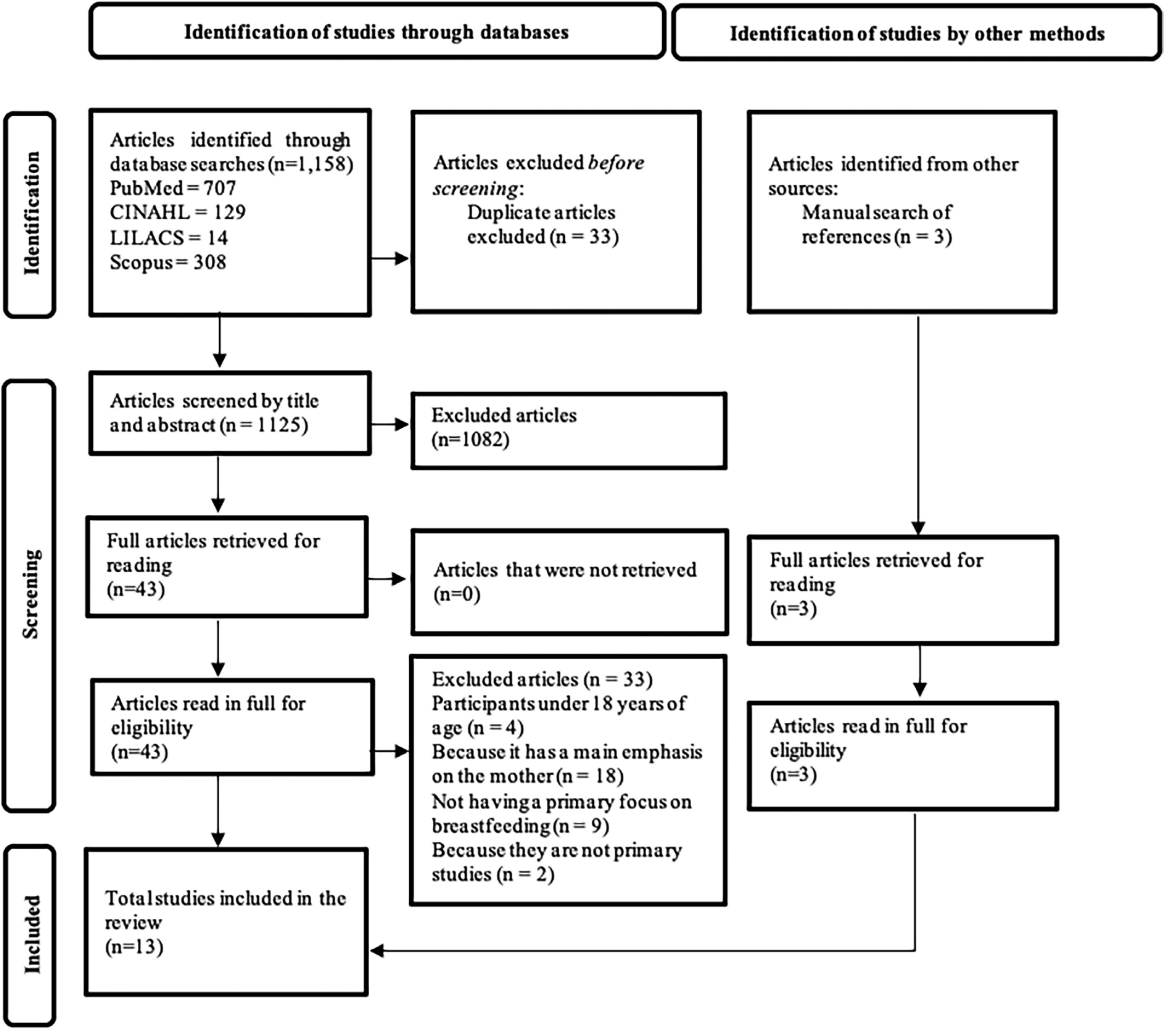
PRISMA flowchart of the literature search process^(24)^.

A total of 1,158 studies were identified in the aforementioned databases and three studies in the manual search, found in the reference list of the included studies, as illustrated in Figure 1, which followed the PRISMA recommendations to describe the literature search process^(24)^. Of these, 33 duplicate articles were excluded, with 1,125 being left. Then, the titles and abstracts were read independently by two reviewers, and the inclusion and exclusion criteria were followed. The articles reviewed by the two authors were combined, resulting in 43 articles that met the eligibility criteria. The full reading of these articles then began. Of these, 33 were excluded, as they had fathers under 18 years of age (n = 4), the main emphasis on the mother (n = 18), breastfeeding not as their main focus (n = 9), or because they were not primary studies (n = 2). Thus, the final sample of this meta-synthesis consisted of 13 articles (Figure 1).

The quality of the articles was analyzed by two independent reviewers, using CASP (*Critical Appraisal Skills Program*) - an instrument consisting of a checklist for evaluating qualitative studies^(25)^. It contains 10 questions that assess the description and relevance of the objective, the adequacy of the qualitative methodology, the study design, the recruitment strategy, the data collection, the adequacy of the relationship between researcher and participant, the ethical considerations, the rigor of the data analysis, the presentation of the results and the contributions of the study. Disagreements between evaluators were discussed and resolved in face-to-face meetings to reach consensus. The evaluators chose not to exclude the studies for reasons related to methodological quality and the contributions of their results to the theme, which is under construction on the global picture^(26)^.

The included studies were read in full by two independent authors. The data extraction process took place using a form, prepared based on the guiding question of this meta-synthesis and the general characteristics of the studies: year of publication; authors; country of origin; study area; objectives and methodological aspects (study design, participants and data collection procedures). The form was also based on previous qualitative systematic reviews^(27,28)^.

### Data Analysis

The thematic synthesis approach was adopted to analyze and synthesize the data^(21)^. This approach is a three-stage process for theme identification and development, which involves the process of coding text and creating analytical and descriptive themes. The qualitative studies results were coded inductively, line by line, according to their meanings and contents. Similar codes were grouped into a hierarchical tree structure. Soon after, descriptive themes were developed and later interpreted to produce analytical themes to answer the review’s research question. This coding process was done manually and, to ensure the integrity of the codes, two authors coded the data, which were later reviewed by a third author with experience in qualitative research, to recheck the codes consistency and validation. Divergences were discussed among all researchers and consensus was reached^(29)^. Finally, the coding structures were refined until new themes were sufficiently described to explain the analytical themes.

## RESULTS

The studies included in this metasynthesis involved seven countries: Brazil (n = 7), United States (n = 1), Pakistan (n = 1), England (n = 1), United Kingdom (n = 1) Chile (n = 1), and Argentina (n = 1). Most adopted semi-structured interviews for data collection. The 13 studies included a total of 360 fathers participating, with the smallest study including seven fathers and the largest 117, aged 18 to 58 years. Their education level ranged from high school to higher education, and their children were breastfeeding, aged between zero and two years. Regarding cohabitation with the child, 11 studies included only fathers who lived with their wife and child, 1 study included fathers who had daily contact with their children but did not live with them, and 1 study included biological or non-biological fathers of the child, present for most of the breastfeeding process of their partners. Detailed information on the included studies is provided in Chart 2.

**Chart 2:** Characteristics of primary qualitative studies (n = 13) – Teresina, PI, Brazil, 2023.

First author, year and country	Objective	Method	Characterization of fathers
Aguilera-Diaz, 2023Chile^(30)^	To describe the father’s perception of the paternal role in exclusive breastfeeding in the Ñuble region, Chile, during the years 2021 and 2022.	Qualitative study, with phenomenological design. Remote semi-structured interview. The analysis followed the open, axial and selective coding process.	N = 7, age = 26 to 44 years. Biological or non-biological fathers of the child, present for most of the breastfeeding process of their partners, who were studying or had finished higher education.
Azevedo, 2016Brazil^(31)^	To identify the father’s knowledge about breastfeeding.	Qualitative, exploratory, and descriptive study. Data collection through semi-structured interviews, with subsequent content analysis, according to Bardin.	N = 15, Age = 19–51 years. Men with children between seven days and 11 months old. Two had finished higher education; seven had finished high school; two had unfinished high school, and four had unfinished primary education. Eight lived in a common-law marriage and seven were married.
Brown, 2014United Kingdom^(32)^	To explore fathers’ experiences of supporting their partner during breastfeeding and to examine their attitudes towards the education, information and support they have received.	Simple descriptive-qualitative approach study. Online questionnaire, consisting of open questions. Content analysis was performed for each interview through reading to identify emerging themes.	N = 117, age not specified. Fathers whose partners had given birth in the last 2 years and initiated breastfeeding at birth.
Canton, 2022Argentina^(33)^	To explore the bonds that surround breastfeeding mothers based on the value of lactation expressed in their partners reports, in a private maternity hospital in the city of Buenos Aires.	Qualitative study, which used Grounded Theory. Data collection from four focus groups with fathers. Data were processed by defining free-flowing analysis units, coded in two plans: an open coding, in categories that emerged and forming the grouping of categories into five main themes.	N = 16, age = 26 to 41 years. Fathers of medium-high socioeconomic status, with high school or higher education, with Argentinian nationality, two of whom being foreigners.
Fazio, 2018Brazil^(34)^	To identify the structure and contents of the father’s social representation of feeding and exclusive breastfeeding and to analyze the structural relationships between these representations.	Descriptive study, with a qualitative approach, based on the Theory of Social Representations. Data collection took place through free evocations and semi-structured interviews. The analysis adopted context unit coding.	N = 54, age = 18 to 50 years 23 fathers had finished/unfinished primary education, 24 finished/unfinished high school and seven finished/unfinished higher education. Fifty-one lived with their partner and 3 did not live with their partner. All had children and 15 had at least one child from previous relationships.
Gutmann, 2018Brazil^(35)^	To identify the father’s contribution to breastfeeding and newborn care.	Exploratory, descriptive study with a qualitative approach. Data collected through semi-structured interviews. Content analysis was used, according to Bardin.	N = 30, age = 18–50. The majority had finished/unfinished high school and lived with their partner.
Lima, 2020Brazil^(36)^	To identify the father’s perception of breastfeeding.	Descriptive study. Semi-structured interviews, in person or by telephone, with subsequent content analysis, according to Bardin.	N = 11, age = 21 to 26 years. Fathers with finished elementary education, self-reported white, family income of 1 minimum wage, who reported participation in prenatal consultations.
Merritt, 2019England^(37)^	To explore fathers’ beliefs, attitudes and behaviors regarding breastfeeding and how they positively or negatively impact their partners’ decisions to start or continue breastfeeding.	Descriptive study, of a qualitative nature. Individual interview with fathers, who may be accompanied by their partners. Analysis by the constant comparative method.	N = 18, age = 21 to 42 years, average 33.6 years. All participants classified themselves as ‘White – British’ except four, who identified themselves as ‘White – Other’. The majority had a university degree (n = 13). Only one classified himself as unemployed. Four interviewees worked for the Armed Forces.
Mitchell-Box, 2012United States^(38)^	To explore male partner perceptions of breastfeeding to inform the development of interventions to increase their support for breastfeeding.	Qualitative study, using grounded theory. Semi-structured interviews were analyzed using the Charmaz technique.	N = 14, age = 20–45 years.A convenience sample of 14 male partners of low-income pregnant or postpartum women. Fathers expecting a child or who have a child under 1 year old.
Mithani, 2015Pakistan^(39)^	To explore fathers’ perceptions of breastfeeding their babies.	Exploratory study, with a qualitative approach. Semi-structured interviews were conducted. Thematic analysis was carried out through six stages of the iterative process.	N = 12, with unspecified age, with six participants (fathers) from the urban area and six (fathers) from the semi-urban area. Furthermore, in the urban sample, fathers were older compared to those in the semi-urban environment.
Pinto, 2018Brazil^(40)^	Understand paternal perception regarding difficulties in breastfeeding.	Descriptive research, with a qualitative approach, through semi-structured interviews. Data were analyzed using the Collective Subject Discourse technique.	N = 12, age 30 to 28 years. Fathers who had children and the mother/child binomial had some difficulty in breastfeeding. All fathers lived with their families, eight of whom were married and four in a common-law marriage. Most of them finished high school and all of them had paid jobs.
Resende, 2014Brazil^(41)^	To present the feelings reported by fathers and their contributions to the breastfeeding process.	Qualitative study, of the phenomenological type. Interviews were conducted with a guiding question. The analysis of the points obtained in these profiles was carried out in conjunction with the list of contributions reported by the fathers.	N= 40, Age = 19–58 years. Fathers with children who are breastfeeding from 6 months to 2 years. Fathers whose partners were breastfeeding.
Teston, 2018Brazil^(42)^	To understand how the father receives his role in relation to breastfeeding.	Descriptive study, of a qualitative nature. Data collected through interviews with one guiding question and two supporting questions. Data were subjected to content analysis, thematic modality.	N = 14, age = 18–35, average 30 years. Seven attended the current birth, 57.1% were from the municipality where the institution under study was located; 42.8% were white, 35.71% were mixed race, and 21.42% were black; 82% had at least six years of education.

Source: own authorship.

The quality of the studies included in the metasynthesis was satisfactory (Chart 3). All of them presented objectives, methodology, and results clearly, as well as a recruitment strategy to achieve the research objectives. The majority, however, did not report the relationship between researcher and participant. Only three articles partially described the ethical issues used and three partially described the contributions of the research to practice.

**Chart 3:** Result of the quality assessment of articles (n = 13) – Teresina, PI, Brazil, 2024.

(First author, year)	Items									
	1	2	3	4	5	6	7	8	9	10
Aguilera-Díaz, 2023^(30)^	✓	✓	✓	✓	✓	✓	✓	✓	✓	✓
Azevedo, 2016^(31)^	✓	✓	✓	✓	✓	X	✓	✓	✓	X
Brown, 2014^(32)^	✓	✓	✓	✓	✓	✓	X	X	✓	✓
Canton, 2022^(33)^	✓	✓	✓	✓	✓	✓	✓	✓	✓	✓
Fazio, 2018^(34)^	✓	✓	✓	✓	✓	X	✓	✓	✓	✓
Gutmann, 2018^(35)^	✓	✓	✓	✓	✓	X	✓	X	✓	✓
Lima, 2020^(36)^	✓	✓	✓	✓	✓	X	✓	✓	✓	✓
Merritt, 2019^(37)^	✓	✓	✓	✓	✓	X	X	✓	✓	X
Mitchell-Box, 2012^(38)^	✓	✓	✓	✓	✓	✓	✓	✓	✓	X
Mithani, 2015^(39)^	✓	✓	✓	✓	X	X	X	X	✓	✓
Pinto, 2018^(40)^	✓	✓	✓	✓	✓	X	✓	✓	✓	✓
Resende, 2014^(41)^	✓	✓	✓	✓	✓	X	✓	✓	✓	✓
Teston, 2018^(42)^	✓	✓	✓	✓	✓	✓	✓	✓	✓	✓

Source: own authorship.

Note: ✓ – Yes; X – Partially Reported or Not.

1. Were the research objectives clearly stated?

2. Was the qualitative methodology appropriate?

3. Was the research design adequate to achieve the proposed objectives?

4. Was the recruitment strategy appropriate to the research objectives?

5. Were the data collected in a way that addressed the research question?

6. Was the relationship between the researcher and participants properly considered?

7. Have ethical issues been considered?

8. Was the data analysis rigorous enough?

9. Were the results reported clearly?

10. Does the research bring contributions?

### Thematic Summary

The analysis of the 13 articles led to the creation of two analytical themes, which qualify the paternal influence on the decision to maintain or interrupt breastfeeding: ‘Difficulty in actively participating in the breastfeeding process’, and ‘Acting directly or indirectly with the mother during breastfeeding’ (Chart 4).

**Chart 4:** Analytical and descriptive themes that qualify paternal influence in the decision to maintain or interrupt breastfeeding – Teresina, PI, Brazil, 2024.

Analytical themes	Descriptive themes
Difficulty in actively participating in the breastfeeding process	– Supporting role in the decision to breastfeed; – Helplessness and suffering in the face of breastfeeding difficulties; – (Un)aware of the breastfeeding benefits; – Feeling excluded in the breastfeeding process.
Interacting directly or indirectly with the mother during breastfeeding	– Encourages breastfeeding; – Support for the use of breast milk substitutes; – Embarrassment due to partner breastfeeding in public.

Source: own authorship.

### Difficulty in Actively Participating in the Breastfeeding Process

In the first analytical theme, the results describe the role played by the father in the decision to maintain or interrupt breastfeeding, which allowed identifying their knowledge about breastfeeding and their feeling of impotence, suffering, and exclusion in this process.

#### Supporting role in the decision to breastfeed

Studies have shown that the father assumes a supporting role in the decision-making process of maintaining or interrupting breastfeeding, acting in a secondary role in which, sometimes, he abstains from this decision. Some studies have shown unconditional support for the partner in this regard, as they believe that the mother knows her limits better and knows what decision to make^(37,38)^. At times, the father refrains from helping her make this decision, as he understands his role as secondary in the breastfeeding process and considers this to be an exclusive decision for the mother^(37,38)^. In other studies, there was evidence of a lack of discussion of this subject by some couples, also due to the belief that the mother knows what is best, since it is her body^(30)^. “She deals with the pain of breastfeeding and spends a lot of time breastfeeding, which becomes tiring for the mother: It is the wife, the woman, who has to deal with the pain of breastfeeding, how it hurts, and the woman who has to actually decide”^(38)^.

On the other hand, the father expects the mother to breastfeed, as he understands that it is the best food to nourish the child, and this perception is based on information acquired during pregnancy^(32)^. Some studies have reported situations in which the mother did not want to breastfeed, and the man questioned her about this decision^(38)^.

This way, concern for the child’s nutrition also leads the father to exercise vigilance over breastfeeding^(32)^. “She gets nervous because she puts him to the breast, and then he is sleeping, with his still mouth, and she says he is sucking, but with his mouth not moving? Then I say: I don’t think so! Then she told me to go away. I want to make sure, isn’t he hungry? Because he doesn’t know how to speak yet”^(42)^.

#### Helplessness and suffering in the face of breastfeeding difficulties

Faced with the mother’s difficulties in the breastfeeding process, the fathers reported concern, sadness, nervousness, and a feeling of helplessness and frustration^(40)^. They claimed not to know how to help the partners^(37,40)^. Therefore, they expressed negative feelings regarding the couple during the breastfeeding period and were worried about not knowing more information on the subject^(30)^. They tried to encourage them not to give up and suffered together when faced with problems or difficulties related to breastfeeding, such as pain when the baby latched on^(40)^. They also exposed the difficulties faced by women during the process of adapting to breastfeeding^(38)^. They reported that it was difficult to see their partners struggling to breastfeed and not being able to do anything, feeling guilty when their partner felt pain, such as when the baby bit the mother’s breast^(32)^. Some reported having purchased an intermediate nipple to help the baby breastfeed, preventing the mother from feeling pain^(40)^. The pain felt by the mother is what helped the fathers understand that breastfeeding was not easy^(40,42)^. In addition to the baby crying, the mother feels pain in her belly and breastfeeding positions are not comfortable^(42)^.

Fathers wanted to help in this process, taking care of household chores or seeking information on what they should do to help their wives^(35)^. However, some felt upset and helpless because they wanted to help with the breastfeeding process but didn’t know how to do it. According to them, sometimes ­professionals did not validate the fathers’ feelings^(32)^. “When I told a nurse I felt helpless, she said enjoy your break and laughed, as if it had nothing to do with me”^(32)^.

Men, in general, consider that the couple is not prepared to deal together with the difficulties arising from breastfeeding and believe that the best strategy is to prepare by expecting the worst experience^(37)^. They stated that this experience is different for each child^(35)^. They also reported that, given the difficulties they encountered, their partners sometimes showed signs of exhaustion, crying and becoming nervous^(31,32,42)^. At that time, they sought guidance from health professionals on the correct latch, to avoid pain during breastfeeding^(31,32,42)^.

#### (Un)aware of the breastfeeding benefits

The majority stated that the best food for their child is breast milk and reported knowing this even before attending prenatal consultations^(37)^. In the studies analyzed, they demonstrated that they know that breast milk changes its composition throughout lactation, providing different nutrients and components that are suitable for each stage of the child’s development^(32,36)^. “It is a complete food and has all the necessary nutrients that the baby needs. It’s the best food there is! Even more so that it becomes strong, gains resistance, right?!”^(40)^. They also reported believing that breast milk is the best milk, as it helps the child to become stronger, preventing diseases, in addition to helping fathers save money^(31,36,39)^. Therefore, they believe that it should be the child’s main food until 6 months^(31)^, and that breastfeeding has to continue until two years of age^(31,41)^.

However, some mentioned not understanding the benefits of breastfeeding, only knowing that it is important for the baby’s health^(31,36)^. They claimed not to have received guidance during prenatal care. “Nobody talked about breastfeeding during prenatal appointments”; “I don’t remember anyone giving any guidance about breastfeeding”^(42)^. “I haven’t received any information and I didn’t even go after it to find out more”^(40)^.

There were also discrepancies regarding the guidelines given to fathers by different health professionals. In this sense, they would like professionals to provide the same guidance, as they feel confused amidst the whirlwind of different guidelines^(37,42)^. Others reported feelings of exclusion, even when participating in prenatal care: “The information was all geared towards my wife. What she could eat, do, try, etc. I know she was the key player here, but I felt like it had nothing to do with me”^(32)^.

#### Feeling excluded in the breastfeeding process

Some fathers reported that they missed having the same bond of mothers and the baby, as they believed that the woman already has a natural bond with her child^(37)^. “Because the mother already has a natural connection. What is pregnancy, nine months with her, everything develops there with her, you know? The father is just a spectator. Does he watch?”^(31)^.

Although some find the bond between mother and child beautiful, they felt excluded from the breastfeeding moment ^(32,37)^. “I know I shouldn’t have felt this way, but until my wife started mixed feeding I… I did, and I know again I shouldn’t have felt this way, but I felt removed from the situation”^(37)^.

### Interacting Directly or Indirectly with the Mother During Breastfeeding

The second analytical theme describes the father’s participatory actions as an encourager of breastfeeding or breast milk substitutes and discomfort with breastfeeding in public.

#### Breastfeeding encouragement

The man encourages the breastfeeding process by providing support to both mother and child^(36)^. This encouragement can be done through gestures, such as making both of them comfortable and providing a calm and peaceful environment so that the mother can breastfeed comfortably. Such paternal attitudes make the father a fundamental part of the breastfeeding process, assuming the role of support so that the mother does not give up and remains firm in this purpose^(35,40)^. For some, the paternal role in breastfeeding demands patience, as they often feel quite tired and, even so, need to hold the baby, take the child to breastfeed, then put him/her in the crib, several times a night^(31)^. In the studies analyzed, some fathers stated that they considered themselves important in this breastfeeding process^(30)^. “Yes, I was important […] Not physically, but in terms of monitoring, etc.”^(30)^ Some report not being present because they work a lot and this makes it difficult for them to participate^(31)^.

With all these paternal demands during breastfeeding, fathers believed that paternity leave could facilitate participation in breastfeeding, since they could take care of the other children and some household chores, in addition to being with the woman at all times^(39,41)^. These fathers also believed they could help by seeking instructions on the main care for the baby and the mother, as well as equipping themselves with strategies to transform the home into a calm and welcoming environment^(41)^. One participant reported being next to the mother and cuddling the baby while breastfeeding^(30)^. “I would usually also approach him and touch his forehead or cheek, to also participate in the act”^(30)^.

Some believed that fighting domestic violence that they had previously practiced against their partners before the birth of their children was also a strategy to support breastfeeding^(39)^. “I always support my wife to breastfeed... I never hurt her. Even when I want to hit her I don’t because I know she is very fragile”^(39)^.

On the other hand, some fathers felt that they were unable to play any direct role in breastfeeding, as they considered it to be the mother’s sole responsibility to promote breastfeeding. For them, the role they assumed was simply to support the family, as this way the mother would not need to work and would be available to the baby at any time^(31,39)^. They also reported the desire for their wives to remain at home to maintain the breastfeeding process^(33)^. “It would be great if she stayed home and I worked… but traditional gender roles cannot be achieved economically”^(33)^.

#### Support for the use of breast milk substitutes

Some fathers believed that breast milk was not enough to nourish their children^(34)^ and that the formula should be used, as it is a sufficient food for the baby’s nutritional needs^(32)^. According to them, the use of breast milk substitutes was healthier, as well as more convenient, with the child being able to gain more weight^(39)^. Some even said they did not notice any differences between breast milk and formula, as, for them, both had the same benefits for nourishing their children and, therefore, they chose to use formula^(32)^.

In some cases, the use of formula was described as bringing several benefits, such as practicality in feeding the child, the freedom to leave the house more often with the partner and the perception of contributing to the baby’s longer sleep^(38)^. Fathers also wanted to be more involved in breastfeeding and therefore reported that they felt more connected to their children when they bottle-fed them^(32,37)^. Another benefit they mentioned was taking turns feeding the baby, as the use of milk substitutes allowed them to help their partners in this task, which could not be done with exclusive breastfeeding^(38)^.

Some, in order to feel more included during the feeding process of their children, chose to introduce the bottle, as they wanted to feel useful and included in the breastfeeding process^(32,35)^. With this, they felt that they were giving some kind of pleasure and care to their children, in addition to actions such as changing diapers and bathing them^(37)^. Furthermore, they reported understanding the importance of paternal presence during the breastfeeding process and throughout their children’s growth^(30,37)^. According to them, because they missed their fathers when they were little, they wanted to be more present in their children’s lives^(33)^.

Other studies, on the other hand, described fathers’ understanding that formula was unhealthy for their children, as it could cause diarrhea and malnutrition^(39)^. In this sense, fathers referred to this food as an artificial product, capable of causing harm to the child, in addition to being a source of soy^(30)^. Another disadvantage mentioned was the high cost, as the baby ingests a lot of milk in the first six months of life^(32,39,40,41)^. Therefore, many prefer that their wives continue breastfeeding, as this helps them save money^(32,40)^.

#### Embarrassment due to partner breastfeeding in public

Some fathers felt uncomfortable seeing their partners breastfeeding in front of friends and family, even though it was something natural, as they associated breastfeeding with something sexual or perverted^(32,38)^. Some asked their partners to try to hide their breasts: “Just try, hide it as much as you can, I guess”^(38)^. Many preferred women to breastfeed in the presence of strangers rather than with acquaintances, as they believed that it would be more embarrassing for anyone watching^(32,38)^. Others didn’t like her breastfeeding in front of her older children, as they believed they were no longer old enough to see their own mother’s breasts^(38)^.

## DISCUSSION

This review allowed us to synthesize qualitative scientific evidence on the father’s influence on the decision to continue or interrupt breastfeeding. Two analytical themes were developed, which highlight the father’s knowledge about breastfeeding, its influence on the decision-making process about continuing or stopping breastfeeding, and the feelings and difficulties they experienced during breastfeeding. This meta-synthesis also identified how fathers feel about breastfeeding in public and which interconnected factors interfere in this process, influenced by masculinity, which is equally affected throughout this process.

The results corroborate other reviews, in which the partner’s involvement during breastfeeding can contribute to the maintenance or interruption of this process, as well as broaden the approach to the difficulties faced by fathers during this period, such as lack of information and fear^(43)^.

The father’s involvement can promote breastfeeding, as he can help with household chores and take care of other children, so that the mother can breastfeed. In addition, when he has access to information relevant to breastfeeding, he can encourage his wife and thus favor breastfeeding continuation^(18)^. Even verbalized support from the partner is associated with increased duration and exclusivity of breastfeeding^(19)^. Fathers’ recognition that breast milk is the most effective and nutritious food for a baby’s development also helps them encourage their partners to continue breastfeeding, despite the possible difficulties they may face^(44)^.

The paternal experience regarding breastfeeding, however, is also permeated by several ambiguous feelings, as they feel happy and want to support breastfeeding, at the same time as they feel frustrated and excluded^(45)^. This is due, among other reasons, to the final decision of whether or not to breastfeed being exclusively made by the mother, with fathers feeling excluded because they do not know how to participate in this process^(14,17,45,46)^. Research shows that fathers feel some limitations in supporting their partners in the breastfeeding process, including being present during breastfeeding due to social constructions related to masculinity^(47)^.

Studies report that attention to fathers is reduced or non-existent during prenatal care and, as a result, they end up not receiving sufficient information about breastfeeding, in addition to some not continually attending consultations^(45)^. Research also shows the need for more educational strategies to involve fathers during the breastfeeding period^(48,49)^.

The father’s involvement in prenatal care is extremely important, as at this time he receives information about the benefits of breast milk and can help and encourage his partner during the breastfeeding process^(18)^.

This monitoring can alleviate the psychological and emotional impact, as they begin to assume new family roles, as father and mother, and access to information allows them to become aware that they can play an important role in breastfeeding, strengthening the father-mother-child family bond^(50)^. On the other hand, it is noted that some strategies are required that reach fathers who claim not to have time to do this monitoring, or who do not show interest in monitoring, as access to information can directly interfere with their opinion on breastfeeding^(11,18)^.

With the arrival of a child, the family routine changes, including the couple’s sex life, as the mother needs to pay more attention to the newborn, which is why many fathers feel excluded from daily life. To deal with this situation, some help with breastfeeding, while others prefer to stay away from this process, arguing that they are not the ones breastfeeding^(44)^.

This is the first qualitative metasynthesis to systematically review the literature on paternal influence on breastfeeding decision-making. The rigor employed in the method of this review, which used the SPIDER tool, and the inclusion of four databases complemented with manual searches are strengths of this investigation. Since the last decade, there has been an increase in the encouragement of paternal participation in care activities, including support for breastfeeding^(4)^. However, the number of studies in the final sample, which reflects recent trends on the topic, may be a limitation of this review, which opens up perspectives for future studies in high, middle, and low-income countries, and highlights the need for more research focused on paternal influence in the breastfeeding decision-making process and on fathers’ participation during breastfeeding as a whole.

In general, fathers receive little information about breastfeeding and feel excluded or do not know how to participate in this process. In this context, this meta-synthesis helps health professionals, especially nurses, to involve the father since prenatal care to identify informational and emotional needs related to breastfeeding, to establish support interventions in favor of the father’s self-efficacy and inclusion, as well as the decision-making role and encouragement to maintain breastfeeding. This will help alleviate fathers’ fears and anxiety, as well as contribute to the continuation of exclusive breastfeeding.

## CONCLUSION

This review allowed the analysis and synthesis of knowledge published in the last 10 years about the father’s influence on the decision to maintain or interrupt child’s breastfeeding. It is a complex experience, in which the man begins to assume a new role, that of being a father, and this generates fear and apprehension. The decision to stop or continue breastfeeding is usually made by the mother, and the father supports this decision. The father also feels excluded from this process, as well as confused about what he should do to help his partner and child.

The father was represented with great importance during breastfeeding, and can help throughout this process when he has access to information, especially during prenatal care, as this guidance eases the dilemma of knowing how to participate and be included during breastfeeding. Although they see breastfeeding as a painful and difficult experience for their partners, they consider breast milk the best food for their children. Furthermore, although some help with household chores and other tasks to facilitate the breastfeeding process for their partners, some do not know how to participate in the breastfeeding process and feel excluded. In this sense, the father can and should be included in the breastfeeding process, and health professionals have to include him since prenatal care, to favor the bond between husband and wife, as well as the continuity of breastfeeding.

## References

[1] Carvalho LMN, Passos SG (2021). Os benefícios do aleitamento materno para a saúde da criança: revisão integrativa. Rev Coleta Científica.

[2] Quigley MA, Kelly YJ, Sacker A (2019). Breastfeeding and hospitalization for infectious illnesses in early childhood: evidence from the millennium cohort study. Pediatrics.

[3] Aoyama EA, Silva EP, Silva ET (2020). A importância do aleitamento materno nos seis primeiros meses de vida do recém-nascido. Rev Bras Interdiscip Saúde.

[4] World Health Organization. (2009). Baby-friendly hospital initiative: revised, updated and expanded for integrated care [Internet]..

[5] World Health Organization. (2023). Breastfeeding [Internet]..

[6] Neves PAR, Vaz JS, Maia FS, Baker P, Gatica-Domínguez G, Piwoz E (2021). Rates and time trends in the consumption of breastmilk, formula, and animal milk by children younger than 2 years from 2000 to 2019: analysis of 113 countries. Lancet Child Adolesc Health.

[7] Suliman O, Alsharif WM, Alsaedi EA, Alhazmi LS, Reshwan LM, Alharbi NN (2023). The effect of weaning practices on the nutritional and health status of saudi preschool children. Cureus.

[8] North K, Gao M, Allen G, Lee ACC (2022). Breastfeeding in a global context: epidemiology, impact, and future directions. Clin Ther.

[9] Lackey KA, Fehrenkamp BD, Pace RM, Williams JE, Meehan CL, McGuire MA (2021). Breastfeeding beyond 12 months: is there evidence for health impacts? Annu Rev Nutr. 2021;41(1):283–308. doi: http://doi.org/10.1146/annurev-nutr-043020-011242. 2021;41(1):283–308.

[10] Wagner LPB, Mazza VA, Souza SRRK, Chiesa A, Lacerda MR, Soares L (2020). Strengthening and weakening factors for breastfeeding from the perspective of the nursing mother and her family. Rev Esc Enferm USP.

[11] Nagel EM, Howland MA, Pando C, Stang J, Mason SM, Fields DA (2022). Maternal psychological distress and lactation and breastfeeding outcomes: a narrative review. Clin Ther.

[12] Alves YR, Couto LL, Barreto ACM, Quitete JB (2020). Breastfeeding under the umbrella of support networks: a facilitative strategy. Esc Anna Nery.

[13] Agrawal J, Chakole S, Sachdev C (2022). The role of fathers in promoting exclusive breastfeeding. Cureus.

[14] Ogbo FA, Akombi BJ, Ahmed KY, Rwabilimbo AG, Ogbo AO, Uwaibi NE (2020). Breastfeeding in the community: how can partners/fathers help? A systematic review. Int J Environ Res Public Health.

[15] Chang YS, Li KMC, Li KYC, Beake S, Lok KYW, Bick D (2021). Relatively speaking? partners’ and family members; views and experiences of supporting breastfeeding: a systematic review of qualitative evidence. Philos Trans R Soc Lond B Biol Sci.

[16] Wynter K, Di Manno L, Watkins V, Rasmussen B, Macdonald JA (2021). Midwives’ experiences of father participation in maternity care at a large metropolitan health service in Australia. Midwifery.

[17] Budiati T, Setyowati S, Adjie S, Gunawijaya J (2022). Fathers’ role in sustainability of exclusive breastfeeding practice in post-cesarean-section mothers. J Public Health Res.

[18] Bráulio TIC, Damasceno SS, Cruz RSBLC, Figueiredo MFER, Silva JMFL, Silva VM (2021). Conhecimento e atitudes paternas acerca da importância do aleitamento materno. Esc Anna Nery.

[19] Ogbo FA, Akombi BJ, Ahmed KY, Rwabilimbo AG, Ogbo AO, Uwaibi NE (2020). Amamentação na comunidade – Como os parceiros/pais podem ajudar? Uma revisão sistemática. Int J Environ Res Public Health.

[20] Walsh D, Downe S (2005). Meta-synthesis method for qualitative research: a literature review. J Adv Nurs.

[21] Thomas J, Harden A (2008). Methods for the thematic synthesis of qualitative research in systematic reviews. BMC Med Res Methodol.

[22] Tong A, Flemming K, McInnes E, Oliver S, Craig J (2012). Enhancing Transparency in Reporting the Synthesis of Qualitative Research: ENTREQ. BMC Med Res Methodol.

[23] Methley AM, Campbell S, Chew-Graham C, McNally R, Cheraghi-Sohi S (2014). PICO CSS. PICOS and SPIDER: a comparison study of specificity and sensitivity in three search tools for qualitative systematic reviews.

[24] Page MJ, McKenzie JE, Bossuyt PM, Boutron I, Hoffmann TC, Mulrow CD (2021). The PRISMA 2020 statement: an updated guideline for reporting systematic reviews. BMJ.

[25] Critical Appraisal Skills Programme – CASP. (2013). Making sense of evidence: 10 questions to help you make sense of qualitative research [Internet]..

[26] Carroll C, Booth A (2015). Quality assessment of qualitative evidence for systematic review and synthesis: is it meaningful, and if so, how should it be performed?. Res Synth Methods..

[27] Alvarenga WA, Silva SS, Resende MR, Santos GN (2014). Fatores determinantes e condicionantes para o sobrepeso e a obesidade em pré-escolares: uma revisão integrativa. Rev Interd.

[28] Leite ACAB, Garcia-Vivar C, Neris RR, Alvarenga WA, Nascimento LC (2019). The experience of hope in families of children and adolescents living with chronic illness: a thematic synthesis of qualitative studies. J Adv Nurs.

[29] Bradley EH, Curry LA, Devers KJ (2007). Qualitative data analysis for health services research: developing taxonomy, themes, and theory. Health Serv Res.

[30] Aguilera-Díaz J, Castillo-Oyarzo I, García-Yáñez C, Garrido-Muñoz K, Gelabert RC, Aedo BC (2023). Percepción del padre de su rol hacia la lactancia materna en la región de Ñuble, Chile, 2021. Rev Obstet Ginecol Venez.

[31] Azevedo SJS, Santos FAPS, Vieira CENK, Mariz LS, Silva NA, Enders BC (2016). Knowledge of man about breastfeeding / Conhecimento do homem sobre aleitamento materno. Acta Sci Health Sci.

[32] Brown A, Davies R (2014). Fathers’ experiences of supporting breastfeeding: challenges for breastfeeding promotion and education. Matern Child Nutr.

[33] Canton C, Baston C, Álvarez Gatti P, Vecchiarelli C, Osio C (2022). Perspectivas y valoración de la lactancia en los padres varones de una maternidad privada de la Ciudad Autónoma de Buenos Aires: investigación cualitativa. Arch Argent Pediatr.

[34] Fazio IA, Silva CD, Acosta DF, Mota MS (2018). Alimentação e aleitamento materno exclusivo do recém nascido: representação social do pai. Rev Enferm UERJ.

[35] Gutmann VLR, Silva CD, Fazio HA, Mota MS, Acosta DF (2018). Cuidados com o recém-nascido: contribuição do pai no aleitamento materno. Vittalle Rev Ciênc Saúde.

[36] Lima WC, Castro MR, Santos EFO, Calado HSA, Malkes NFA, Wanderley TC (2020). A percepção do pai sobre o aleitamento materno. Braz J Health Rev.

[37] Merritt R, Vogel M, Ladbury P, Johnson S (2019). A qualitative study to explore fathers’ attitudes towards breastfeeding in south West England. Prim Health Care Res Dev.

[38] Mitchell-Box K, Braun KL (2012). Fathers’ thoughts on breastfeeding and implications for a theory-based intervention. J Obstet Gynecol Neonatal Nurs.

[39] Mithani Y, Premani ZS, Kurji Z, Rashid S (2015). Exploring fathers’ role in breastfeeding practices in the urban and semiurban settings of Karachi, Pakistan. J Perinat Educ.

[40] Pinto KRTF, Martins JR, Campos MC, Quintamilha TDF, Zani AV, Bernardy CCF (2018). Dificuldades na amamentação: sentimentos e percepções paternas. J Nurs Health.

[41] Resende TC, Dias EP, Cunha CMC, Mendonça GS, Ribeiro AL, Santos LRL (2014). Participação paterna no período da amamentação: importância e contribuição. Biosci J.

[42] Teston EF, Reis TS, Góis LM, Spigolon DN, Maran E, Marcon SS (2018). Aleitamento materno: percepção do pai sobre seu papel. Rev Enferm Centro-oeste Min.

[43] Constant RJS, Mariot MDMC (2020). Percepção paterna sobre o aleitamento materno: uma revisão integrativa. RevCuidado Enferm-Cesuca.

[44] Marques LF, Ribeiro RV, Amaral JBM (2019). Marques LF, Ribeiro RV, Amaral JBM. O papel da figura paterna na manutenção do aleitamento materno: revisão integrativa Integrative Review Rev Intellectus. 2019;59:5–15.. Marques LF, Ribeiro RV, Amaral JBM. O papel da figura paterna na manutenção do aleitamento materno: revisão integrativa Integrative Review Rev Intellectus. 2019;59:5–15..

[45] Watkins V, Kavanagh SA, Macdonald JA, Rasmussen B, Maindal HT, Hosking S (2024). “I always felt like I wasn’t supposed to be there”. An international qualitative study of fathers’ engagement in family healthcare during transition to fatherhood.

[46] Bráulio TIC, Gomes EB, Matos JHF, Oliveira CJ, Alencar AMPG, Callou RSBL (2022). Influência paterna no aleitamento materno: uma revisão de escopo. Rev Renome.

[47] Martínez-Plascencia U, Rangel-Flores Y, Rodríguez-Martinez ME (2017). ¿Lactancia materna o en pareja? Un estudio sobre las experiencias de reconfiguración de cuerpos, roles y cotidianeidades en madres y padres mexicanos. Cad Saude Publica.

[48] Abbass-Dick J, Dennis CL (2018). Maternal and paternal experiences and satisfaction with a co-parenting breastfeeding support intervention in Canada. Midwifery.

[49] Jeong J, Sullivan EF, McCann JK (2023). Effectiveness of father-inclusive interventions on maternal, paternal, couples, and early child outcomes in low- and middle-income countries: a systematic review. Soc Sci Med.

[50] Lopes GS, Sousa TV, Freitas DA, Carvalho Fa FSS, Sá ES, Vasconcelos AC (2021). Os benefícios do pré-natal masculino para a consolidação do trinômio mãe-pai-filho: uma revisão integrativa. Rev Divulgação Científica Sena Aires.

