# Supplementary material for: Father's influence on breastfeeding continuity or interruption: meta-synthesis
Source: Rev Esc Enferm USP. 2025 Apr 11;59:e20240303. doi: 10.1590/1980-220X-REEUSP-2024-0303en (PMC12004997; doi:10.1590/1980-220X-REEUSP-2024-0303en)
Supplement: Translation

# Translation: Influência do pai na continuidade ou interrupção do aleitamento materno: metassíntese

**DOI:** 10.1590/1980-220X-REEUSP-2024-0303pt

**Authors:** Willyane de Andrade Alvarenga, Thais Rayane da Conceição Gomes, Carlos Henrique Rodrigues da Silva, Giovanna Cristina Machado Kayzuka, Francine de Montigny, Adriana Moraes Leite, Lucila Castanheira Nascimento

**Affiliations:** 1 Centro Universitário Santo Agostinho, Teresina, PI, Brasil.; 2 Universidade de São Paulo, Escola de Enfermagem de Ribeirão Preto, Ribeirão Preto, SP, Brasil.; 3 Université du Québec en Outaouais, Département des sciences infirmières, Gatineau, Canada.

**Keywords:** Pai, Desmame precoce, Aleitamento, Pesquisa qualitativa

## Abstract

**Objetivo:**

Sintetizar evidências qualitativas sobre a influência do pai na decisão da mãe de manter ou interromper o aleitamento materno.

**Método:**

Metassíntese qualitativa, com buscas sistemáticas nas bases de dados LILACS, PubMed, Scopus e CINAHL, suplementadas por buscas manuais. Foram seguidas as recomendações do ENTREQ (Enhancing Transparency in Reporting the Synthesis of Qualitative Research) e o checklist CASP (Critical Appraisal Skills Programme) para avaliar a qualidade dos estudos. No processo de síntese dos dados, utilizou-se abordagem de síntese temática.

**Resultados:**

De um total de 1.158 referências identificadas, 13 foram incluídas. A síntese temática possibilitou a construção de dois temas analíticos, que qualificam a influência paterna: ‘Dificuldade em se inserir ativamente no processo de amamentação’, com quatro temas descritivos, e ‘Contracenando direta ou indiretamente com a mãe na amamentação’, com três temas descritivos.

**Considerações Finais:**

A maioria dos estudos revelou o processo decisório centrado na mãe. Educação em saúde direcionada ao pai durante o pré-natal contribui para uma paternidade participativa e incentivadora do aleitamento materno.

## INTRODUÇÃO

O leite materno é um alimento completo, de suma importância para o desenvolvimento nutricional, neurológico e imunológico do bebê^(1)^. É capaz de reduzir a incidência de mortalidade infantil por doenças preveníveis, por auxiliar no fortalecimento do sistema imune^(2)^. Crianças amamentadas exclusivamente até os seis meses de idade apresentam menor incidência de internação hospitalar, principalmente por diarreia^(3)^. Dessa forma, a Organização Mundial da Saúde preconiza a manutenção do aleitamento materno em livre demanda de forma exclusiva até os seis meses de vida^(4)^. Após esse período, orienta-se introduzir alimentação complementar, porém mantendo a amamentação de maneira complementar até os dois anos ou mais^(5)^.

Apesar dos benefícios do aleitamento materno para o lactente, permanece baixo o índice de mulheres que amamentam seus filhos exclusivamente, pois muitas optam pelo uso de suplementos, como as fórmulas infantis^(6)^. Por consequência do desmame precoce, as crianças podem desenvolver problemas de saúde, como diarreias agudas, anemias, déficits nutricionais, maior sensibilidade em adquirir doenças crônicas, como a diabetes, infecções e doenças respiratórias, como bronquite e asma, além de aumento do risco de morte por falta de nutrientes^(7)^. Dentre os fatores que contribuem para o desmame precoce, estão dificuldades relacionadas à falta de orientação para amamentação, necessidade de retornar ao trabalho antes dos seis meses do bebê, sofrimento psicológico e ausência de rede de apoio para manutenção do aleitamento materno^(8-11)^.

A disponibilidade de uma rede de apoio, formada por familiares, amigos ou profissionais da saúde, mostra-se essencial para o fortalecimento de aspectos físicos, sociais, psicológicos, culturais e profissionais da mulher^(12)^. No geral, tal suporte é representado por outras mulheres do círculo de convivência, como mãe, sogra ou tias, principalmente nos primeiros meses após o parto^(12)^. O contato com o histórico e vivências de amamentação dessas mulheres, além de fornecer ferramentas para superar dificuldades frequentes neste período, interfere na manutenção do aleitamento materno por essas mães^(12,13)^.

A presença do parceiro enquanto rede de apoio, no entanto, não é clara na literatura. Enquanto algumas evidências sublinham a baixa disponibilidade paterna em participar da amamentação^(12)^, outras mostram que estes representam a maior rede de apoio de puérperas^(13)^. A ausência do apoio paterno prejudica a qualidade do aleitamento materno, sobretudo pelo acúmulo de tarefas domiciliares por essas mães, o que acarreta sentimentos de solidão, desistência de seguimento médico e crença de que o leite é insuficiente para nutrição de seus filhos^(12,13)^. Dessa forma, o apoio do parceiro é reconhecido como um divisor de águas na decisão de manter a amamentação, além de auxiliar na criação de um modelo familiar focado no bem-estar e no fortalecimento dos vínculos^(13,14)^.

Assim, a ausência de apoio paterno e os consequentes problemas familiares são fatores que interferem no sucesso aleitamento materno^(15)^. O reconhecimento do pai e de sua importância no período gravídico contribui para o crescimento saudável da criança, amamentação e puerpério da parceira^(13,16)^. O pai, inclusive, mostra-se como sendo o principal agente para a busca de maneiras para ajudar e confortar a esposa/parceira^(17)^, principalmente durante o aleitamento materno^(17,18)^. No entanto, ainda não é claro na literatura como ele influencia a decisão materna de manter ou interromper o aleitamento do filho^(19)^, tampouco há revisões de literatura de estudos qualitativos na perspectiva paterna.

Diante do exposto e da importância de melhor investigar essa temática, esta pesquisa objetivou sintetizar evidências qualitativas sobre a influência do pai na decisão da mãe de manter ou interromper o aleitamento materno. Para tanto, definiu-se a seguinte questão de pesquisa: “Quais as evidências qualitativas sobre a experiência paterna na decisão sobre a continuidade ou interrupção do aleitamento materno?”.

## MÉTODO

### Tipo de Estudo

Trata-se de uma metassíntese qualitativa, que consiste em uma análise ampla de estudos com abordagem qualitativa, contribuindo para a compreensão de um problema particular^(20)^. Para a síntese dos dados, utilizou-se a abordagem proposta por Thomas e Harden^(21)^ da síntese temática dos estudos qualitativos, conforme as seguintes etapas: busca por estudos relevantes; avaliação da qualidade dos estudos qualitativos; extração dos dados; e síntese temática. Recomendações descritas no ENTREQ (*Enhancing Transparency in Reporting the Synthesis of Qualitative Research*) foram utilizadas para relatar esta metassíntese qualitativa^(22)^.

### Local, População e Critérios De Seleção

A busca foi realizada por dois revisores, independentemente, para levantamento dos estudos primários nas bases de dados: PubMed, Scopus, CINAHL e LILACS. Realizou-se ainda busca manual nas listas de referências dos artigos incluídos. Selecionaram-se estudos nos idiomas português, espanhol e inglês pela fluência do grupo de pesquisa responsável, com o período de publicação de janeiro de 2012 a dezembro de 2023. O período selecionado garante que a revisão inclua dados atualizados, alinhados com as práticas atuais e políticas de saúde de incentivo à participação paterna no aleitamento materno, que foram reflexos da introdução da Iniciativa Hospital Amigo da Criança, iniciada pela Organização Mundial de Saúde (OMS) e pela UNICEF^(4)^. Esse recorte temporal permite uma revisão focada em pesquisas que refletem a evolução dessas diretrizes e abordagens.

Os diferentes descritores e as palavras-chaves relacionados a ‘pai/*father*’, ‘desmame/*Weaning*’ e ‘amamamentação/*Breast Feeding*’ foram selecionados de acordo com os Descritores em Ciências da Saúde (DeCS) e do *Medical Subject Headings* (MeSH) e combinados com o uso dos operadores Booleanos (AND, OR). Utilizou-se a ferramenta SPIDER (amostra, fenômeno de interesse, design, avaliação e tipo de pesquisa) para estruturar a estratégia de busca a ser empregada na base de dados Pubmed (Quadro 1) e adaptada a outras bases de dados, conforme as especificidades de cada uma^(23)^.

**Quadro 1:** Termos de busca empregados na base de dados Pubmed – Teresina, PI, Brasil, 2024.

SPIDER	DESCRIÇÃO	TERMOS DE BUSCA (DeCs, MeSH e palavras-chaves)
*Sample* (Amostra)	Pai	“Fathers”[Mesh] OR “Fathers” OR ”Father” OR “Spouses”[Mesh] OR “Spouses” OR “Spouse”
*Phenomenon of Interest* (Fenômeno de interesse)	Aleitamento	(“Breast Feeding”[Mesh] OR (Breastfed) OR (Breastfeeding)
*Design* (Desenho)	Métodos e técnicas de estudos qualitativos	“Focus Groups”[Mesh] OR (Focus Group) OR (Group, Focus) OR “Anthropology, Medical”[Mesh] OR (Medical Anthropology) OR “Grounded Theory”[Mesh] OR (Theory, Grounded) OR Culture OR (Thematic synthesis) OR “Hermeneutics”[Mesh] OR (Hermeneutic) OR (Ethnographic) OR (ethnographic research) OR (Phenomenology) OR (phenomenological research) OR (Narrative) OR “Interviews as Topic”[Mesh] OR (Interviewers) OR (Interviewer) OR (Interviewees) OR (Group Interviews) OR (Group Interview) OR (Interview, Group) OR (Interviews, Group) OR (in-depth interview) OR (qualitative interview) OR (content analysis) OR (semantic analysis)
*Evaluation* (Resultado)	Desmame precoce	“Weaning”[Mesh] OR (weaning)
*Research Type* (Tipo de pesquisa)	Pesquisa qualitativa	“Qualitative Research”[Mesh] OR (Research, Qualitative) OR (Qualitative studies) OR (Qualitative) OR “Empirical Research”[Mesh] OR (Research, Empirical)

Fonte: Autoria própria.

Foram incluídos estudos primários, de abordagem qualitativa, sobre a experiência paterna durante o período de aleitamento materno. Os artigos deveriam ter o pai/homem como participante e seriam incluídos estudos com amostras mistas caso os resultados referentes ao pai fossem apresentados e analisados separadamente dos demais participantes. Estudos de métodos mistos, em que os resultados qualitativos estivessem apresentados e analisados separadamente dos dados quantitativos, também seriam incluídos. Foram excluídos estudos de revisão de literatura, pesquisas quantitativas, editoriais, resumos de congressos, teses e dissertações e estudos com participantes menores de 18 anos ou que apresentassem conjuntamente a experiência do pai, mãe ou profissional de saúde, de forma indiscriminada.

### Coleta de Dados

Identificou-se um total de 1.158 estudos nas referidas bases de dados e três estudos na busca manual, encontrados na lista de referências dos estudos incluídos, conforme ilustrado na Figura 1, a qual seguiu as recomendações PRISMA para descrever o processo de busca na literatura^(24)^. Destes, foram excluídos 33 artigos duplicados, restando 1.125. Em seguida, realizou-se a leitura dos títulos e resumos por dois revisores, de forma independente, observando-se os critérios de inclusão e exclusão. Procedeu-se com a junção dos artigos revisados pelos dois autores, sendo obtidos 43 artigos que se adequaram aos critérios de elegibilidade. Iniciou-se, então, a leitura na íntegra desses artigos, dos quais 33 foram excluídos, por terem pais menores de 18 anos (n = 4), ênfase principal na mãe (n = 18), não terem como foco principal o aleitamento materno (n = 9) e não serem estudos primários (n = 2). Assim, a amostra final desta metassíntese foi constituída por 13 artigos (Figura 1).

**Figura 1:** Fluxograma PRISMA do processo de busca na literatura^(24)^.

A qualidade dos artigos foi analisada por dois revisores independentes, sendo utilizado o CASP (*Critical Appraisal Skills Programme*) - instrumento que consiste em um *checklist* para avaliação de estudos qualitativos^(25)^. Ele contém 10 questões que avaliam a descrição e relevância do objetivo, a adequação da metodologia qualitativa, o delineamento do estudo, a estratégia de recrutamento, a coleta de dados, a adequação da relação entre pesquisador e participante, as considerações éticas, o rigor da análise dos dados, a apresentação dos resultados e as contribuições do estudo. As discordâncias entre os avaliadores foram discutidas e resolvidas em reuniões presenciais para obtenção de consenso. Os avaliadores optaram pela não exclusão dos estudos por motivos relacionados à qualidade metodológica e pelas contribuições de seus resultados para a temática, a qual se encontra em construção no cenário mundial^(26)^.

Os estudos incluídos foram lidos na íntegra por dois autores independentes. O processo de extração dos dados ocorreu por meio de um formulário, elaborado com base na questão norteadora desta metassíntese e nas características gerais dos estudos: ano de publicação; autores; país de origem; área do estudo; objetivos e aspectos metodológicos (delineamento do estudo, participantes e procedimentos para coleta de dados). O formulário também foi baseado em revisões sistemáticas qualitativas prévias^(27,28)^.

### Análise dos Dados

A abordagem de síntese temática foi adotada para analisar e sintetizar os dados^(21)^. Esta abordagem é um processo de três estágios para a identificação e desenvolvimento do tema, que envolve o processo de codificação de texto e criação de temas analíticos e descritivos. Os resultados dos estudos qualitativos foram codificados indutivamente, linha por linha, de acordo com seus significados e conteúdos. Códigos semelhantes foram agrupados em uma estrutura de árvore hierárquica. Logo após, desenvolveram-se temas descritivos, posteriormente interpretados para produzir temas analíticos a fim de responder à questão de pesquisa da revisão. Esse processo de codificação foi feito manualmente e, para garantir a integridade dos códigos, dois autores codificaram os dados, os quais foram revisados posteriormente por um terceiro autor com experiência em pesquisa qualitativa, para verificar novamente a consistência e validação dos códigos. Divergências foram discutidas entre todos os pesquisadores e obteve-se consenso^(29)^. Por fim, as estruturas de codificações foram refinadas até que novos temas fossem suficientemente descritos para explicar os temas analíticos.

## RESULTADOS

Os estudos incluídos nesta metassíntese envolveram sete países: Brasil (n = 7), Estados Unidos (n = 1), Paquistão (n = 1), Inglaterra (n = 1), Reino Unido (n = 1) Chile (n = 1) e Argentina (n = 1). A maioria adotou entrevistas semiestruturadas para coleta de dados. Os 13 estudos incluem um total de 360 pais participantes, com o menor estudo incluindo sete pais e o maior 117, com idades entre 18 e 58 anos. A escolaridade variou entre ensino médio e ensino superior completos, e seus filhos estavam em período de amamentação, com idades de zero a dois anos. Quanto à coabitação com o filho, 11 estudos incluíram somente pais que residiam com a esposa e o filho, 1 estudo incluiu pais que conviviam com os filhos diariamente, mas não residiam com eles e 1 estudo incluiu pais biológicos ou não biológicos da criança, presentes na maior parte do processo de amamentação das companheiras. O detalhamento das informações sobre os estudos incluídos está no Quadro 2.

**Quadro 2:** Características dos estudos qualitativos primários (n = 13) – Teresina, PI, Brasil, 2023.

Primeiro autor, ano e país	Objetivo	Método	Caracterização dos pais
Aguilera-Díaz, 2023 Chile^(30)^	Descrever a percepção do pai sobre o papel paterno na amamentação exclusiva na região de Ñuble, Chile, durante os anos de 2021 e 2022.	Estudo qualitativo, com desenho fenomenológico. Entrevista semiestruturada remota. A análise seguiu o processo de codificação aberta, axial e seletiva.	N = 7, idade = 26 a 44 anos. Pais biológicos ou não biológicos da criança, presentes na maior parte do processo de amamentação das companheiras, que estivessem cursando ou concluído o ensino superior.
Azevedo, 2016 Brasil^(31)^	Identificar o conhecimento do pai sobre o aleitamento materno.	Estudo qualitativo, exploratório e descritivo. Coleta de dados por meio de entrevistas semiestruturadas, com posterior análise conteúdo, segundo Bardin.	N = 15, Idade = 19–51 anos. Homens com crianças entre sete dias e 11 meses. Dois tinham ensino superior completo; sete ensino médio completo; dois não concluíram o ensino médio e quatro não concluíram o ensino fundamental. Oito viviam em união estável e sete eram casados.
Brown, 2014 Reino Unido^(32)^	Explorar experiências dos pais de apoio à parceira durante a amamentação e examinar suas atitudes para a educação, informação e apoio que elas têm recebido.	Estudo de abordagem descritiva-qualitativa simples. Questionário on-line, composto de perguntas abertas. A análise de conteúdo foi executada para cada entrevista mediante leitura para identificação de temas emergentes.	N = 117, idade não especificada. Pais cujas parceiras tivessem dado à luz nos últimos 2 anos e iniciado a amamentação ao nascer.
Canton, 2022 Argentina^(33)^	Explorar os vínculos que cercam as mães que amamentam a partir da valorização da lactação manifestada no relato de seus companheiros, em uma maternidade privada da cidade de Buenos Aires.	Estudo qualitativo, que utilizou a Teoria Fundamentada nos Dados. Coleta de dados a partir de quatro grupos focais com pais. Os dados foram processados definindo unidades de análise de fluxo livre, codificadas em dois planos: uma codificação aberta, em categorias que emergiram e formando o agrupamento das categorias em cinco temas principais.	N = 16, idade = 26 a 41anos. Pais de nível socioeconômico médio-alto, com ensino médio ou ensino superior, com nacionalidade argentina, sendo dois estrangeiros.
Fazio, 2018 Brasil^(34)^	Identificar a estrutura e os conteúdos da representação social do pai acerca da alimentação e do aleitamento materno exclusivo e analisar as relações estruturais entre essas representações.	Estudo descritivo, com abordagem qualitativa, fundamentado na Teoria das Representações Sociais. A coleta de dados ocorreu por meio de evocações livres e entrevistas semiestruturadas. A análise adotou a codificação da unidade de contexto.	N = 54, idade = 18 a 50 anos 23 pais possuíam ensino fundamental completo/incompleto, 24 ensino médio completo/incompleto e sete ensino superior completo/incompleto. 51 moravam com a companheira e 3 não residiam com a companheira. Todos tinham filhos e 15 tinham pelo menos um filho de relacionamentos anteriores.
Gutmann, 2018 Brasil^(35)^	Identificar a contribuição do pai no aleitamento materno e nos cuidados com o recém-nascido.	Estudo exploratório, descritivo, com abordagem qualitativa. Dados coletados por entrevista semiestruturada. Utilizou-se a análise de conteúdo, segundo Bardin.	N = 30, idade = 18–50. A maioria possuía ensino médio completo/incompleto e morava com a companheira.
Lima, 2020 Brasil^(36)^	Identificar a percepção do pai diante do aleitamento materno.	Estudo descritivo. Entrevistas semiestruturadas, presenciais ou por telefone, com posterior análise de conteúdo, segundo Bardin.	N = 11, idade = 21 a 26 anos. Pais com ensino fundamental completo, prevalência étnica autorreferida branca, renda familiar de 1 salário mínimo, que referiam participação nas consultas de pré-natal.
Merritt, 2019 Inglaterra^(37)^	Explorar as crenças, atitudes e comportamentos dos pais em relação à amamentação e como eles impactam positiva ou negativamente nas decisões de suas parceiras para iniciar ou continuar amamentando.	Estudo descritivo, de natureza qualitativa. Entrevista individual com os pais, podendo estar acompanhados por suas parceiras. Análise pelo método comparativo constante.	N = 18, idade = 21 a 42 anos, média de 33,6 anos. Todos os participantes se classificaram como ‘Brancos - britânicos’, exceto quatro, que se identificaram como ‘Brancos – Outro’. A maioria possuía diploma universitário (n = 13). Apenas um se classificou como desempregado. Quatro entrevistados trabalhavam para as Forças Armadas.
Mitchell-Box, 2012 Estados Unidos^(38)^	Explorar as percepções do parceiro masculino sobre amamentação para informar o desenvolvimento de intervenções para aumentar seu apoio à amamentação.	Estudo qualitativo, usando a teoria fundamentada. Entrevistas semiestruturadas foram analisadas pela técnica de Charmaz.	N = 14, idade = 20–45 anos. Uma amostra de conveniência de 14 parceiros do sexo masculino de gestantes ou puérperas de baixa renda. Pais esperando um filho ou que tivessem uma criança menor de 1 ano.
Mithani, 2015 Paquistão^(39)^	Explorar as percepções dos pais sobre a amamentação de bebês.	Estudo exploratório, de abordagem qualitativa. Conduzidas entrevistas semiestruturadas. A análise temática foi realizada por meio de seis etapas do processo iterativo.	N = 12, com idade não especificada, sendo seis participantes (pais) da zona urbana e seis (pais) da zona semiurbana. Além disso, na amostra urbana, os pais eram mais velhos em comparação com os do meio semiurbano.
Pinto, 2018 Brasil^(40)^	Compreender a percepção paterna diante das dificuldades no aleitamento materno.	Pesquisa descritiva, com abordagem qualitativa, por meio de entrevistas semiestruturadas. Os dados foram analisados utilizando a técnica do Discurso do Sujeito Coletivo.	N = 12, idade 30 a 28 anos. Pais que possuíam filhos nascidos e que o binômio mãe/filho apresentava alguma dificuldade na amamentação. Todos os pais residiam com suas famílias, sendo oito casados e quatro em união estável. A maioria concluiu o ensino médio e todos exerciam atividade remunerada.
Resende, 2014 Brasil^(41)^	Apresentar os sentimentos referidos por pais e suas contribuições no processo de aleitamento materno.	Estudo qualitativo, do tipo fenomenológico. Foram realizadas entrevistas com questão norteadora. A análise dos pontos obtidos nesses perfis foi feita em conjunto com a listagem das contribuições relatadas pelos pais.	N = 40, Idade = 19–58 anos. Pais com filhos em período de amamentação de 6 meses a 2 anos. Pais cujas companheiras estivessem em período de aleitamento materno.
Teston, 2018 Brasil^(42)^	Compreender de que modo o pai recebe seu papel em relação ao aleitamento materno.	Estudo descritivo, de natureza qualitativa. Dados coletados por entrevista com uma questão norteadora e duas questões de apoio. Os dados foram submetidos à análise de conteúdo, modalidade temática.	N = 14, idade = 18–35, média de 30 anos. Sete acompanharam o parto atual, 57,1% eram do município de localização da instituição em estudo; 42,8% da raça branca, 35,71% pardos e 21,42% negros; 82% possuíam, no mínimo, seis anos de estudo.

Fonte: autoria própria.

A qualidade dos estudos incluídos na metassíntese foi satisfatória (Quadro 3). Todos apresentaram objetivo, metodologia e resultados de maneira clara, assim como estratégia de recrutamento para o alcance dos objetivos da pesquisa. A maioria, contudo, não informou a relação entre pesquisador e participante. Somente três artigos descreveram parcialmente as questões éticas utilizadas e três parcialmente as contribuições da pesquisa para a prática.

**Quadro 3:** Resultado da avaliação da qualidade dos artigos (n = 13) – Teresina, PI, Brasil, 2024.

(Primeiro autor, ano)	Itens									
	1	2	3	4	5	6	7	8	9	10
Aguilera-Díaz, 2023^(30)^	✓	✓	✓	✓	✓	✓	✓	✓	✓	✓
Azevedo, 2016^(31)^	✓	✓	✓	✓	✓	X	✓	✓	✓	X
Brown, 2014^(32)^	✓	✓	✓	✓	✓	✓	X	X	✓	✓
Canton, 2022^(33)^	✓	✓	✓	✓	✓	✓	✓	✓	✓	✓
Fazio, 2018^(34)^	✓	✓	✓	✓	✓	X	✓	✓	✓	✓
Gutmann, 2018^(35)^	✓	✓	✓	✓	✓	X	✓	X	✓	✓
Lima, 2020^(36)^	✓	✓	✓	✓	✓	X	✓	✓	✓	✓
Merritt, 2019^(37)^	✓	✓	✓	✓	✓	X	X	✓	✓	X
Mitchell-Box, 2012^(38)^	✓	✓	✓	✓	✓	✓	✓	✓	✓	X
Mithani, 2015^(39)^	✓	✓	✓	✓	X	X	X	X	✓	✓
Pinto, 2018^(40)^	✓	✓	✓	✓	✓	X	✓	✓	✓	✓
Resende, 2014^(41)^	✓	✓	✓	✓	✓	X	✓	✓	✓	✓
Teston, 2018^(42)^	✓	✓	✓	✓	✓	✓	✓	✓	✓	✓

Fonte: autoria própria.

Nota: ✓ – Sim; X – Parcialmente Reportado ou Não.

1. Os objetivos da pesquisa estavam reportados claramente?

2. A metodologia qualitativa foi adequada?

3. O desenho da pesquisa estava adequado para alcance dos objetivos propostos?

4. A estratégia de recrutamento foi adequada aos objetivos da pesquisa?

5. Os dados foram coletados de modo que abordassem a questão de pesquisa?

6. A relação entre o pesquisador e os participantes foi devidamente considerada?

7. As questões éticas foram consideradas?

8. A análise dos dados foi suficientemente rigorosa?

9. Os resultados foram reportados claramente?

10. A pesquisa traz contribuições?

### Síntese Temática

A análise dos 13 artigos conduziu a elaboração de dois temas analíticos, as quais qualificam a influência paterna na decisão de manter ou interromper o aleitamento materno: ‘Dificuldade em se inserir ativamente no processo de amamentação’ e ‘Contracenando direta ou indiretamente com a mãe na amamentação’ (Quadro 4).

**Quadro 4:** Temas analíticos e descritivos que qualificam a influência paterna na decisão de manter ou interromper o aleitamento materno – Teresina, PI, Brasil, 2024.

Temas analíticos	Temas descritivos
Dificuldade em se inserir ativamente no processo de amamentação	– Papel coadjuvante na decisão pela amamentação; – Impotência e sofrimento diante das dificuldades da amamentação; – (Des)Conhecedor dos benefícios do aleitamento materno; – Sentir-se excluído no processo da amamentação.
Contracenando direta ou indiretamente com a mãe na amamentação	– Incentivar do aleitamento materno; – Apoio ao uso dos substitutos do leite materno; – Constrangimento pela companheira amamentar em público.

Fonte: autoria própria.

### Dificuldade em se inserir ativamente no processo de amamentação

No primeiro tema analítico, os resultados descrevem o papel desempenhado pelo pai quanto à decisão de manter ou interromper a amamentação, o que possibilitou identificar o conhecimento destes a respeito do aleitamento materno e o sentimento de impotência, sofrimento e exclusão nesse processo.

#### Papel coadjuvante na decisão pela amamentação

Os estudos demonstraram que o pai assume um papel coadjuvante no processo decisório de manter ou interromper a amamentação, atuando em um papel secundário em que, algumas vezes, abstém-se desta decisão. Alguns estudos evidenciaram apoio incondicional à parceira neste quesito, por acreditarem que a mãe conhece melhor os seus limites e sabe qual decisão tomar^(37,38)^. Em alguns momentos, o pai se abstém de auxiliá-la nesta tomada de decisão, pois entende o seu papel como secundário no processo da amamentação e considera esta uma decisão exclusiva da mãe^(37,38)^. Em outros estudos, evidenciou-se ausência de discussão desse assunto por alguns casais, também pela crença de que a mãe sabe o que é o melhor, uma vez que se trata do corpo dela^(30)^. “Ela lida com a dor da amamentação e passa muito tempo amamentando, o que se torna cansativo para a mãe: É a esposa, mulher, que tem que lidar com a dor da amamentação, como dói, e a mulher que tem que decidir na verdade”^(38)^.

Por outro lado, o pai espera que a mãe amamente, por entender que é o melhor alimento para nutrir o filho, sendo esta percepção baseada em informações adquiridas durante a gestação^(32)^. Alguns estudos relataram situações em que a mãe não quis amamentar, e o homem a questionou sobre essa decisão^(38)^.

Dessa forma, a preocupação com a nutrição do filho também leva o pai a exercer a vigilância da amamentação^(32)^. “Ela fica nervosa porque ela o põe no peito, aí ele está com a boca parada dormindo, ela diz que ele está mamando, mas com a boca sem mexer? Daí eu falo: Eu acho que não está! Aí ela me mandou sair de perto. Eu quero ter certeza, ele não está passando fome não? Porque ele não sabe falar ainda”^(42)^.

#### Impotência e sofrimento diante das dificuldades da amamentação

Diante de dificuldades da mãe no processo da amamentação, os pais relataram preocupação, tristeza, nervosismo e uma sensação de impotência e frustração^(40)^. Eles afirmaram não saber como ajudá-las^(37,40)^. Por isso, manifestavam sentimentos negativos em relação ao casal durante o período de amamentação e se preocupavam por desconhecerem mais informações sobre o assunto^(30)^. Eles tentavam incentivá-las a não desistir e sofriam juntos diante dos problemas ou dificuldades relacionadas à amamentação, como a dor na pega do bebê^(40)^. Eles também expuseram as dificuldades enfrentadas pelas mulheres durante o processo de adaptação na amamentação^(38)^. Relataram ser difícil ver suas parceiras lutando para amamentar e não poderem fazer nada, sentindo-se culpados quando a companheira sentia dor, como quando o bebê mordia a mama da mãe^(32)^. Alguns relataram ter comprado bico intermediário para ajudar o bebê a mamar, evitando que a mãe sentisse dor^(40)^. A dor sentida pela mãe é o que ajudava na compreensão dos pais de que a amamentação não era fácil^(40,42)^. Além do choro do bebê, a mãe sente dores na barriga e as posições para amamentar não são confortáveis^(42)^.

Os pais desejavam ajudar nesse processo, cuidando dos afazeres domésticos ou buscando informações sobre o que deveriam fazer para auxiliar suas esposas^(35)^. Entretanto, alguns se sentiam chateados e impotentes, pois desejavam ajudar no processo de aleitamento materno, mas não sabiam como fazê-lo. Segundo eles, às vezes, os profissionais não validavam esse sentimento dos pais^(32)^. “Quando eu disse a uma enfermeira que me senti impotente, ela disse aproveite o intervalo e riu, como se não tivesse nada a ver comigo”^(32)^.

Os homens, no geral, consideram que o casal não é preparado para lidar em conjunto com as dificuldades oriundas da amamentação e acreditam que a melhor estratégia é se preparar tendo a expectativa da pior experiência^(37)^. Eles afirmaram que essa vivência é diferente com cada filho^(35)^. Relataram ainda que, diante das dificuldades encontradas, suas companheiras apresentavam, por vezes, sinais de exaustão, chorando e ficando nervosas^(31,32,42)^. Nesse momento, buscavam, com profissionais de saúde, orientações para a pega correta, de forma a evitar as dores durante a amamentação^(31,32,42)^.

#### (DES)conhecedor dos benefícios do aleitamento materno

A maioria afirmou que o melhor alimento para o filho é o leite materno e referiu saber disso mesmo antes de participar das consultas pré-natal^(37)^. Nos estudos analisados, eles demostraram conhecer que o leite materno muda sua composição durante toda a lactância, fornecendo diferentes nutrientes e componentes que se adequam para cada fase de desenvolvimento da criança^(32,36)^. “É um alimento completo e tem todos os nutrientes necessários que o bebê precisa. É o melhor alimento que existe! Ainda mais para ela ficar forte, pegar resistência né?!”^(40)^. Eles também relataram acreditar que o leite materno é o melhor leite, pois ajuda a criança a ficar mais forte, evitando doenças, além de auxiliar os pais a economizarem^(31,36,39)^. Com isso, acreditam que deva ser o principal alimento da criança até os 6 meses^(31)^, e que o bebê precisa continuar sendo amamentado complementarmente até os dois anos de idade^(31,41)^.

No entanto, alguns mencionaram não compreender os benefícios da amamentação, cientes apenas de ser importante para a saúde do bebê^(31,36)^. Eles alegaram não ter recebido orientações durante o pré-natal. “Ninguém falou sobre aleitamento materno nas consultas de pré-natal”; “não me lembro de terem orientado nada sobre aleitamento”^(42)^. “Não tive nenhuma informação e também nem fui atrás para saber mais”^(40)^.

Ocorreram também divergências quanto às orientações repassadas aos pais por diferentes profissionais de saúde. Nesse sentido, gostariam que os profissionais fornecessem as mesmas orientações, pois se sentem confusos em meio ao turbilhão de orientações distintas^(37,42)^. Outros relataram sentimento de exclusão, mesmo participando do pré-natal: “A informação foi toda voltada para minha esposa. O que ela podia comer, fazer, experimentar etc. Eu sei que ela era a peça-chave aqui, mas senti que não tinha nada a ver comigo”^(32)^.

#### Sentir-se excluído no processo da amamentação

Alguns pais referiram sentir falta de ter o mesmo vínculo da mãe com o bebê, pois acreditavam que a mulher já possui, naturalmente, um vínculo com o filho^(37)^. “Porque a mãe já tem uma ligação natural. O que é gravidez, nove meses com ela, tudo se desenvolve lá com ela, né? O pai é apenas um espectador. Ele assiste?”^(31)^.

Apesar de alguns acharem lindo o vínculo entre mãe e filho, sentiam-se excluídos do momento da amamentação^(32,37)^. “Eu sei que não deveria ter me sentido assim, mas até que minha esposa começar a alimentação mista eu... eu me sentia, e sei novamente que não deveria ter me sentido assim, mas me senti afastado da situação”^(37)^.

### Contracenando direta ou indiretamente com a mãe na amamentação

O segundo tema analítico descreve as ações participativas do pai como incentivador do aleitamento materno ou dos substitutos do leite materno e o desconforto com a amamentação em público.

#### Incentivador do aleitamento materno

O homem incentiva o processo de amamentação ao fornecer apoio ao binômio mãe e filho^(36)^. Esse incentivo pode ser realizado através de gestos, como deixar os dois em conforto e proporcionar um ambiente calmo e tranquilo para que a mãe possa amamentá-lo confortavelmente. Tais atitudes paternas configuram o pai como uma parte fundamental no processo de amamentação, assumindo o papel de apoio para que ela não desista e se mantenha firme nesse propósito^(35,40)^. Para alguns, o papel paterno na amamentação demanda paciência, pois, muitas vezes, sentem-se bastante cansados e, mesmo assim, precisam segurar o bebê, levar a criança para amamentar, depois colocá-la no berço, várias vezes por noite^(31)^. Nos estudos analisados, alguns pais afirmaram que se consideravam importantes nesse processo de amamentação^(30)^. “Sim, fui importante […] Não fisicamente, mas na questão do acompanhamento, etc.”^(30)^. Alguns relatam não estar presentes, pois trabalham muito e isso dificulta a sua participação^(31)^.

Com toda essa demanda paterna durante a amamentação, os pais acreditavam que a licença-paternidade poderia facilitar a participação na amamentação, uma vez que poderiam cuidar dos outros filhos e de alguns afazeres da casa, além de acompanhar a mulher em todos os momentos^(39,41)^. Esses pais também acreditavam ajudar buscando instruções acerca dos principais cuidados com o bebê e a mãe, bem como munindo-se de estratégias para transformar a casa em um ambiente tranquilo e acolhedor^(41)^. Um participante relatou estar ao lado da mãe e fazer carinho no bebê durante a amamentação^(30)^. “Geralmente eu também me aproximava dele e tocava sua testa ou bochecha, para também participar do ato”^(30)^.

Alguns acreditavam que lutar contra a violência doméstica antes praticada por eles contra as companheiras antes do nascimento dos filhos também configura-se como uma estratégia de apoio à amamentação^(39)^. “Eu sempre apoio minha esposa para amamentar... Eu nunca a machuquei. Mesmo quando eu quero bater nela eu não faço porque eu sei que ela é muito frágil”^(39)^.

Por outro lado, alguns pais julgavam que não conseguiam desempenhar nenhum papel direto na amamentação, pois consideravam de inteira responsabilidade da mãe a promoção da amamentação. Para eles, o papel que assumiam era apenas o de sustentar a família, pois assim a mãe não precisaria trabalhar e estaria à disposição do bebê a qualquer hora^(31,39)^. Eles também relataram o desejo que suas esposas continuassem em casa para manter o processo de amamentação^(33)^. “Seria ótimo se ela ficasse em casa e eu trabalhasse…, mas os papéis tradicionais de gênero não podem ser alcançados economicamente”^(33)^.

#### Apoio ao uso dos substitutos do leite materno

Alguns pais acreditavam que o leite materno não era suficiente para a nutrição dos filhos^(34)^ e que a fórmula deveria ser utilizada, por ser um alimento suficiente para as necessidades nutricionais do bebê^(32)^. Segundo eles, o uso dos substitutos do leite materno era mais saudável, além de mais conveniente, com a criança conseguindo ganhar mais peso^(39)^. Alguns, inclusive, afirmaram não perceber diferenças entre o leite materno e a fórmula, pois, para eles, ambos possuíam os mesmos benefícios para nutrir seus filhos e, portanto, optaram pelo uso da fórmula^(32)^.

Em alguns casos, o uso da fórmula foi descrito como carreador de diversos benefícios, como a praticidade ao alimentar o filho, a liberdade de sair mais vezes de casa com a companheira e a percepção de contribuir para um sono mais prolongado do bebê^(38)^. Os pais também desejavam ser mais participativos na amamentação e, por isso, relataram que se sentiam mais ligados aos filhos quando os alimentavam com a mamadeira^(32,37)^. Outro benefício citado por eles foi o revezamento na alimentação do bebê, pois o uso dos substitutos do leite possibilitava que auxiliassem suas companheiras nessa tarefa, o que não poderia ser feito com o aleitamento materno exclusivo^(38)^.

Alguns, para se sentirem mais incluídos durante o processo de alimentação de seus filhos, optaram por introduzir a mamadeira, pois queriam se sentir úteis e incluídos no processo de amamentação^(32,35)^. Com isso, sentiram que estavam dando algum tipo de prazer e cuidado aos filhos, para além de ações como trocar fralda e dar banho^(37)^. Além disso, relataram compreender a importância da presença paterna durante o processo de amamentação e ao longo do crescimento de seus filhos^(30,37)^. Segundo eles, por terem sentido a falta dos pais quando pequenos, desejavam estar mais presentes na vida de seus filhos^(33)^.

Outros estudos, por outro lado, descreveram o entendimento paterno de que a fórmula não era saudável para seus filhos, pois poderia causar diarreias e má nutrição^(39)^. Nesse sentido, os pais referiram-se a esse alimento como um produto artificial, capaz de causar malefícios à criança, além de ser uma fonte de soja^(30)^. Outra desvantagem citada foi o alto custo, pois o bebê ingere muito leite nos primeiros seis meses de vida^(32,39-41)^. Dessa forma, muitos preferiram que a suas esposas continuem amamentando, pois isso os ajuda a economizar^(32,40)^.

#### Constrangimento pela companheira amamentar em público

Alguns pais se sentiam desconfortáveis ao verem suas parceiras amamentando na frente dos amigos e familiares, mesmo sendo algo natural, pois relacionavam a mama a algo sexual ou pervertido^(32,38)^. Alguns solicitavam que suas companheiras tentassem esconder a mama: “Apenas tente, esconda-a o máximo que puder, eu acho”^(38)^. Muitos preferiam que a mulher amamentasse na presença de estranhos do que ao lado de conhecidos, pois acreditavam que o constrangimento maior seria para quem estivesse olhando^(32,38)^. Já outros não gostavam ela amamentasse na frente dos filhos mais velhos, pois acreditavam que não estavam mais na idade de ver as mamas da própria mãe^(38)^.

## DISCUSSÃO

Essa revisão permitiu sintetizar evidências científicas qualitativas sobre a influência do pai na decisão de continuar ou interromper o aleitamento materno. Dois temas analíticos foram elaborados, as quais destacam o conhecimento do pai sobre a amamentação, sua influência na tomada de decisão sobre continuar ou interromper o aleitamento e os sentimentos e dificuldades por eles vivenciados durante a amamentação. Essa metassíntese também identificou como os pais se sentem com a amamentação em público e que fatores interligados interferem nesse processo, influenciados pela masculinidade, igualmente afetada ao longo desse percurso.

Os resultados corroboram outras revisões, nas quais o envolvimento do parceiro durante a amamentação pode contribuir para a manutenção ou interrupção desse processo, bem como ampliam a abordagem das dificuldades enfrentadas pelos pais nesse período, como a falta de informação e o medo^(43)^.

O envolvimento do pai pode favorecer a amamentação, uma vez que ele pode auxiliar nos afazeres domésticos e cuidar dos outros filhos, para que a genitora consiga amamentar. Além disso, quando ele tem acesso a informações pertinentes à amamentação, pode encorajar a esposa e, assim, favorecer a continuidade do aleitamento^(18)^. Até mesmo o apoio verbalizado pelo parceiro está associado ao aumento da duração e exclusividade da amamentação^(19)^. O reconhecimento por parte dos pais de que o leite materno é o alimento mais eficaz e nutritivo para o desenvolvimento do bebê também contribui para que incentivem as companheiras a manter a amamentação, apesar das possíveis dificuldades enfrentadas^(44)^.

A experiência paterna acerca do aleitamento materno, no entanto, é também permeada por diversos sentimentos ambíguos, visto que se sentem felizes e querem apoiar a amamentação, ao mesmo tempo em que se sentem frustrados e excluídos^(45)^. Isso se dá, dentre outros motivos, pela decisão final de amamentar ou não ser exclusivamente da mãe, com os pais sentindo-se excluídos por não saberem como participar desse processo^(14,17,45,46)^. Pesquisas afirmam que os pais sentem algumas limitações para apoiar suas companheiras no processo de amamentar, inclusive para estarem presentes na amamentação em virtude das construções sociais relacionadas à masculinidade^(47)^.

Estudos relatam que a atenção aos pais é reduzida ou inexistente durante o pré-natal e, dessa forma, eles acabam não obtendo informações suficientes sobre a amamentação, além de alguns não participarem continuamente das consultas^(45)^. Pesquisas mostram ainda a necessidade de mais estratégias educativas para envolver os pais durante o período de amamentação^(48,49)^.

A inserção do pai no acompanhamento do pré-natal é de suma importância, pois neste momento eles recebem informações sobre os benefícios do leite materno e conseguem auxiliar e incentivar as companheiras durante o processo de amamentação^(18)^.

Esse acompanhamento pode amenizar o impacto psicológico e emocional, pois passam a assumir novos papéis familiares, de pai e mãe, e o acesso às informações possibilita a conscientização de que podem participar de maneira importante da amamentação, fortalecendo o vínculo familiar pai-mãe-filho^(50)^. Por outro lado, percebe-se a necessidade de estratégias que alcancem os pais que alegam não ter tempo para fazer esse acompanhamento, ou que não demonstram interesse em acompanhar, pois o acesso à informação pode interferir diretamente na opinião dos mesmos sobre o aleitamento materno^(11,18)^.

Com a chegada de uma criança, a rotina familiar muda, inclusive a vida sexual do casal, pois a mãe precisa dar mais atenção ao recém-nascido, motivo pelo qual muitos pais se sentem excluídos do convívio diário. Para lidar com essa situação, alguns auxiliam na amamentação, enquanto outros preferem se manter longe deste processo, sob o argumento de não serem eles que amamentam^(44)^.

Esta é a primeira metassíntese qualitativa a revisar sistematicamente a literatura sobre a influência paterna no processo decisório da amamentação. O rigor empregado no método desta revisão, que usou a ferramenta SPIDER, e a inclusão de quatro bases de dados complementado com buscas manuais são fortalezas desta investigação. Desde a última década, há um aumento no incentivo à participação paterna nas atividades de cuidado, incluindo o suporte à amamentação^(4)^. Entretanto, o número de estudos na amostra final, que reflete tendências recentes sobre o tema, pode ser uma limitação desta revisão, que abre perspectiva para estudos futuros em países de alta, média e baixa renda, e sublinha a necessidade de mais pesquisas focadas na influência paterna no processo decisório da amamentação e na participação dos pais durante o aleitamento como um todo.

De modo geral, o pai recebe pouca informação acerca da amamentação e sente-se excluído ou não sabe como ser participativo nesse processo. Nesse contexto, esta metassíntese contribui para que os profissionais de saúde, sobretudo de enfermagem, envolvam o pai desde o pré-natal para identificação de necessidades informacionais e emocionais relacionadas ao aleitamento materno, de modo a estabelecer intervenções de apoio em prol da autoeficácia e inserção do pai, bem como do papel decisório e do estímulo à manutenção do aleitamento materno. Isso possibilitará amenizar os medos e ansiedade dos pais, além de contribuir para a continuidade da amamentação exclusiva.

## CONCLUSÃO

Esta revisão permitiu a análise e síntese do conhecimento publicado nos últimos 10 anos acerca da influência do pai na decisão de manter ou interromper o aleitamento materno do filho. Trata-se de uma experiência complexa, em que o homem passa a assumir um novo papel, o de ser pai, e isso gera receio e medo. A tomada de decisão sobre a interrupção ou continuidade do aleitamento é geralmente da mãe, e o pai a apoia essa decisão. O pai também se sente excluído desse processo, além de confuso em relação ao que deve fazer para auxiliar sua companheira e filho.

O pai foi representado com grande importância durante a amamentação, podendo auxiliar ao longo desse processo quando tem acesso às informações, principalmente no pré-natal, pois essas orientações facilitam o dilema de saber como participar e ser incluído durante a amamentação. Embora a amamentação seja vista por eles como uma experiência dolorosa e difícil para as companheiras, consideram o leite materno o melhor alimento para seus filhos. Além disso, apesar de alguns ajudarem nos afazeres domésticos e em outros quesitos no intuito de facilitar o processo de amamentação de suas companheiras, alguns não sabem como participar do processo de amamentação e se sentem excluídos. Nesse sentido, o pai pode e deve ser incluído no processo de amamentação, e os profissionais de saúde precisam incluí-lo desde o pré-natal, a fim de favorecer o vínculo entre marido e esposa, bem como a continuidade da amamentação.

# Translation: Influencia del padre en la continuidad o interrupción de la lactancia materna: metasíntesis

**DOI:** 10.1590/1980-220X-REEUSP-2024-0303es

**Authors:** Willyane de Andrade Alvarenga, Thais Rayane da Conceição Gomes, Carlos Henrique Rodrigues da Silva, Giovanna Cristina Machado Kayzuka, Francine de Montigny, Adriana Moraes Leite, Lucila Castanheira Nascimento

**Affiliations:** 1 Centro Universitário Santo Agostinho, Teresina, PI, Brasil.; 2 Universidade de São Paulo, Escola de Enfermagem de Ribeirão Preto, Ribeirão Preto, SP, Brasil.; 3 Université du Québec en Outaouais, Département des sciences infirmières, Gatineau, Canada.

**Keywords:** Padre, Destete, Lactancia Materna, Investigación Cualitativa

## Abstract

**Objetivo:**

Sintetizar evidencia cualitativa sobre la influencia del padre en la decisión de la madre de mantener o interrumpir la lactancia materna.

**Método:**

Metasíntesis cualitativa, con búsquedas sistemáticas en las bases de datos LILACS, PubMed, Scopus y CINAHL, complementadas con búsquedas manuales. Se siguieron las recomendaciones de ENTREQ (*Enhancing Transparency in Reporting the Synthesis of Qualitative Research*) y el *Lista de verificación* CASP (*Critical Appraisal Skills Programme*) para evaluar la calidad de los estudios. En el proceso de síntesis de datos se utilizó un enfoque de síntesis temática.

**Resultados:**

De un total de 1.158 referencias identificadas, se incluyeron 13. La síntesis temática permitió la construcción de dos temas analíticos que cualifican la influencia paterna: ‘Dificultad para participar activamente en el proceso de lactancia materna’, con cuatro temas descriptivos, y ‘Actuando directa o indirectamente con la madre durante la lactancia materna’, con tres temas descriptivos.

**Consideraciones Finales:**

La mayoría de los estudios revelaron que el proceso de toma de decisiones se centra en la madre. La educación sanitaria dirigida a los padres durante la atención prenatal contribuye a la paternidad participativa y fomenta la lactancia materna.

## INTRODUCCIÓN

La leche materna es un alimento completo de gran importancia para el desarrollo nutricional, neurológico e inmunológico de los bebés^(1)^. Puede reducir la incidencia de mortalidad infantil por enfermedades prevenibles al contribuir a reforzar el sistema inmunitario^(2)^. Los niños alimentados exclusivamente con leche materna hasta los seis meses tienen una menor incidencia de ingresos hospitalarios, principalmente por diarrea^(3)^. Por ello, la Organización Mundial de la Salud recomienda mantener la lactancia materna exclusiva a demanda hasta los seis meses de edad^(4)^. Después de este periodo, se recomienda introducir la alimentación complementaria, pero manteniendo la lactancia materna hasta que el niño tenga dos años o más^(5)^. A pesar de los beneficios de la lactancia materna para los lactantes, la tasa de mujeres que amamantan exclusivamente a sus hijos sigue siendo baja, ya que muchas optan por utilizar complementos como los preparados para lactantes^(6)^. Como consecuencia del destete precoz, los niños pueden desarrollar problemas de salud como diarrea aguda, anemia, déficits nutricionales, mayor susceptibilidad a adquirir enfermedades crónicas como diabetes, infecciones y enfermedades respiratorias como bronquitis y asma, así como un mayor riesgo de muerte por falta de nutrientes^(7)^. Entre los factores que contribuyen al destete precoz se encuentran las dificultades relacionadas con la falta de orientación sobre la lactancia materna, la necesidad de volver al trabajo antes de que el bebé cumpla seis meses, el malestar psicológico y la ausencia de una red de apoyo para mantener la lactancia^(8-11)^. La disponibilidad de una red de apoyo, formada por familiares, amigos o profesionales sanitarios, es esencial para reforzar los aspectos físicos, sociales, psicológicos, culturales y profesionales de la mujer^(12)^. En general, este apoyo está representado por otras mujeres del círculo familiar, como madres, suegras o tías, especialmente en los primeros meses tras el parto^(12)^. El contacto con la historia y las experiencias de lactancia de estas mujeres, así como proporcionarles herramientas para superar las dificultades frecuentes durante este periodo, influye en el mantenimiento de la lactancia materna por parte de estas madres^(12,13)^. Sin embargo, la presencia de la pareja como red de apoyo no está clara en la literatura. Mientras algunas evidencias enfatizan la baja disposición de los padres a participar en la lactancia^(12)^, otras muestran que ellos representan la mayor red de apoyo para las madres que han dado a luz recientemente^(13)^. La ausencia de apoyo paterno va en detrimento de la calidad de la lactancia, especialmente por la acumulación de tareas domésticas por parte de estas madres, lo que provoca sentimientos de soledad, alejamiento del seguimiento médico y la creencia de que su leche es insuficiente para alimentar a sus hijos^(12,13)^. Así, el apoyo de la pareja es reconocido como un punto de inflexión en la decisión de continuar con la lactancia materna, además de ayudar a crear un modelo familiar centrado en el bienestar y el fortalecimiento de los vínculos^(13,14)^. Así, la falta de apoyo paterno y los problemas familiares resultantes son factores que interfieren en el éxito de la lactancia materna^(15)^. Reconocer al padre y su importancia durante el embarazo contribuye al crecimiento saludable del niño, la lactancia y el puerperio de la pareja^(13,16)^. Incluso se ha demostrado que los padres son los principales agentes para encontrar formas de ayudar y consolar a sus esposas/parejas^(17)^, especialmente durante la lactancia^(17,18)^. Sin embargo, aún no está claro en la literatura cómo influye en la decisión de la madre de mantener o dejar de amamantar a su hijo^(19)^, ni existen revisiones bibliográficas de estudios cualitativos desde la perspectiva del padre.

Teniendo en cuenta lo anterior y la importancia de investigar mejor esta cuestión, esta investigación tuvo como objetivo sintetizar la evidencia cualitativa sobre la influencia del padre en la decisión de la madre de mantener o abandonar la lactancia materna. Para ello, se definió la siguiente pregunta de investigación: «¿Cuál es la evidencia cualitativa sobre la experiencia paterna en la decisión de mantener o interrumpir la lactancia materna?».

## MÉTODO

### Tipo de Estudio

Se trata de una metasíntesis cualitativa, que consiste en un análisis amplio de estudios con enfoque cualitativo, que contribuyen a la comprensión de un problema particular^(20)^. Para sintetizar los datos, se utilizó el abordaje propuesto por Thomas y Harden^(21)^para la síntesis temática de estudios cualitativos, según los siguientes pasos: búsqueda de estudios relevantes; evaluación de la calidad de los estudios cualitativos; extracción de datos; y síntesis temática. Para informar sobre esta metasíntesis cualitativa se utilizaron las recomendaciones descritas en ENTREQ (*Enhancing Transparency in Reporting the Synthesis of Qualitative Research*)^(22)^. *Lugar*,*población y criterios de selección*

La búsqueda de estudios primarios fue realizada por dos revisores, de forma independiente, en las siguientes bases de datos: PubMed, Scopus, CINAHL y LILACS. También se realizó una búsqueda manual en las listas de referencias de los artículos incluidos. Se seleccionaron estudios en portugués, español e inglés debido al dominio del grupo de investigación responsable, con un período de publicación de enero de 2012 a diciembre de 2023. El período seleccionado asegura que la revisión incluya datos actualizados, en consonancia con las prácticas actuales y las políticas de salud para fomentar la participación paterna en la lactancia materna, que se reflejaron en la introducción de la Iniciativa Hospital Amigo del Niño, iniciada por la Organización Mundial de la Salud (OMS) y UNICEF^(4)^. Este marco temporal permite una revisión centrada en la investigación que refleja la evolución de estas directrices y enfoques.

Los diferentes descriptores y palabras clave relacionados con «*padre/paternidad*», «*destete/destete*» y «*lactancia/amamamantamiento*» se seleccionaron de acuerdo con los Descriptores de Ciencias de la Salud (DeCS) y los *Medical Subjects Headings*(MeSH) y se combinaron utilizando operadores booleanos (AND, OR). Se utilizó la herramienta SPIDER (muestra, fenómeno de interés, diseño, evaluación y tipo de investigación) para estructurar la estrategia de búsqueda a utilizar en la base de datos Pubmed (Cuadro 1) y adaptarla a otras bases de datos, según las especificidades de cada una^(23)^.

**Cuadro 1:** Términos de búsqueda utilizados en la base de datos Pubmed Términos de búsqueda utilizados en la base de datos Pubmed – Teresina, PI, Brasil, 2024.

Spider	Descripción	Términos de búsqueda (DeCs, MeSH y palabras llave)
*Sample* (Muestra)	Padre	“Fathers”[Mesh] OR “Fathers” OR ”Father” OR “Spouses”[Mesh] OR “Spouses” OR “Spouse”
*Phenomenon of Interest* (Fenomeno de interés)	Lactancia	(“Breast Feeding”[Mesh] OR (Breastfed) OR (Breastfeeding)
*Design* (Diseño)	Métodos y técnicas de estudios cualitativos	“Focus Groups”[Mesh] OR (Focus Group) OR (Group, Focus) OR “Anthropology, Medical”[Mesh] OR (Medical Anthropology) OR “Grounded Theory”[Mesh] OR (Theory, Grounded) OR Culture OR (Thematic synthesis) OR “Hermeneutics”[Mesh] OR (Hermeneutic) OR (Ethnographic) OR (ethnographic research) OR (Phenomenology) OR (phenomenological research) OR (Narrative) OR “Interviews as Topic”[Mesh] OR (Interviewers) OR (Interviewer) OR (Interviewees) OR (Group Interviews) OR (Group Interview) OR (Interview, Group) OR (Interviews, Group) OR (in-depth interview) OR (qualitative interview) OR (content analysis) OR (semantic analysis)
*Evaluation* (Resultado)	Destete precoz	“Weaning”[Mesh] OR (weaning)
*Research Type* (Tipo de investigación)	Investigación cualitativa	“Qualitative Research”[Mesh] OR (Research, Qualitative) OR (Qualitative studies) OR (Qualitative) OR “Empirical Research”[Mesh] OR (Research, Empirical)

Fuente: Autoría propia.

Se incluyeron estudios primarios con un enfoque cualitativo sobre la experiencia paterna durante el periodo de lactancia. Los artículos debían tener al padre/hombre como participante y se incluyeron estudios con muestras mixtas si los resultados relativos al padre se presentaban y analizaban por separado de los demás participantes. También se incluyeron los estudios de métodos mixtos en los que los resultados cualitativos se presentaban y analizaban por separado de los datos cuantitativos. Se excluyeron las revisiones bibliográficas, las investigaciones cuantitativas, los editoriales, los resúmenes de congresos, las tesis y disertaciones y los estudios con participantes menores de 18 años o que presentaban indiscriminadamente la experiencia de padres, madres o profesionales de la salud de forma conjunta.

### Recopilación de Datos

Se identificaron un total de 1.158 estudios en las bases de datos mencionadas y tres estudios en la búsqueda manual, encontrados en la lista de referencias de los estudios incluidos, como se ilustra en la Figura 1, que siguió las recomendaciones PRISMA para describir el proceso de búsqueda bibliográfica^(24)^. De ellos, se excluyeron 33 artículos duplicados, quedando 1.125. A continuación, dos revisores leyeron de forma independiente los títulos y resúmenes, observando los criterios de inclusión y exclusión. Los artículos revisados por los dos revisores se fusionaron, obteniéndose 43 artículos que cumplían los criterios de elegibilidad. A continuación, se leyeron estos artículos en su totalidad, de los cuales 33 se excluyeron porque tenían padres menores de 18 años (n = 4), se centraban principalmente en la madre (n = 18), no se centraban principalmente en la lactancia materna (n = 9) y no eran estudios primarios (n = 2). Así, la muestra final de esta metasíntesis consistió en 13 artículos (Figura 1).

**Figura 1:** Diagrama de flujo PRISMA del proceso de búsqueda bibliográfica^(24)^.

La calidad de los artículos fue analizada por dos revisores independientes, utilizando el CASP (*Critical Appraisal Skills Programme*) - un instrumento que consiste en una*lista de comprobación*para evaluar estudios cualitativos^(25)^. Contiene 10 preguntas que evalúan la descripción y relevancia del objetivo, la adecuación de la metodología cualitativa, el diseño del estudio, la estrategia de reclutamiento, la recogida de datos, la adecuación de la relación entre investigador y participante, las consideraciones éticas, el rigor del análisis de los datos, la presentación de los resultados y las aportaciones del estudio. Los desacuerdos entre los evaluadores se debatieron y resolvieron en reuniones presenciales para llegar a un consenso. Los evaluadores optaron por no excluir los estudios por razones relacionadas con su calidad metodológica y las aportaciones de sus resultados al tema, que está en construcción en el escenario mundial^(26)^.

Los estudios incluidos fueron leídos íntegramente por dos autores independientes. El proceso de extracción de datos se realizó mediante un formulario basado en la pregunta guía de esta metasíntesis y en las características generales de los estudios: año de publicación; autores; país de origen; campo de estudio; objetivos y aspectos metodológicos (diseño del estudio, participantes y procedimientos de recogida de datos). El formulario también se basó en revisiones sistemáticas cualitativas anteriores^(27,28)^.

### Análisis de Los Datos

Se adoptó el enfoque de síntesis temática para analizar y sintetizar los datos^(21)^. Este enfoque es un proceso de tres etapas para la identificación y el desarrollo de temas, que implica el proceso de codificación del texto y la creación de temas analíticos y descriptivos. Los resultados de los estudios cualitativos se codificaron inductivamente, línea por línea, según su significado y contenido. Los códigos similares se agruparon en una estructura jerárquica de árbol. A continuación, se desarrollaron e interpretaron los temas descriptivos para producir temas analíticos con el fin de responder a la pregunta de investigación de la revisión. Este proceso de codificación se realizó manualmente y, para garantizar la integridad de los códigos, dos autores codificaron los datos, que luego revisó un tercer autor con experiencia en investigación cualitativa para volver a comprobar la coherencia y la validación de los códigos. Se discutieron las divergencias entre todos los investigadores y se llegó a un consenso^(29)^. Por último, se refinaron las estructuras de codificación hasta que los nuevos temas quedaron suficientemente descritos para explicar los temas analíticos.

## RESULTADOS

Los estudios incluidos en esta metasíntesis abarcaron siete países: Brasil (n = 7), Estados Unidos (n = 1), Pakistán (n = 1), Inglaterra (n = 1), Reino Unido (n = 1) Chile (n = 1) y Argentina (n = 1). La mayoría utilizó entrevistas semiestructuradas para la recogida de datos. Los 13 estudios incluyeron un total de 360 padres participantes, de los cuales el estudio más pequeño incluía a siete padres y el más grande a 117, con edades comprendidas entre los 18 y los 58 años. Su nivel de estudios oscilaba entre la enseñanza secundaria y la superior, y sus hijos eran lactantes, con edades comprendidas entre los cero y los dos años. En cuanto a la convivencia con el niño, 11 estudios incluyeron sólo a padres que vivían con su mujer y su hijo, 1 estudio incluyó a padres que vivían a diario con sus hijos pero no convivían con ellos y 1 estudio incluyó a padres biológicos o no biológicos del niño que estaban presentes durante la mayor parte del proceso de lactancia. Los detalles de los estudios incluidos se muestran en lo Cuadro 2.

**Cuadro 2:** Características de los estudios cualitativos primários (n = 13) – Teresina, PI, Brasil, 2023.

Primer autor, año y país	Objetivo	Método	Caracterización de los progenitores
Aguilera-Díaz, 2023 Chile^(30)^	Describir las percepciones de los padres sobre el rol paterno en la lactancia materna exclusiva en la región de Ñuble, Chile, durante los años 2021 y 2022.	Estudio cualitativo con un diseño fenomenológico. Entrevista semiestructurada a distancia. El análisis siguió el proceso de codificación abierta, axial y selectiva.	N = 7, edad = 26 a 44 años. Padres biológicos o no biológicos del niño, presentes durante la mayor parte del proceso de lactancia con sus parejas, que estudiaban o habían completado estudios superiores.
Azevedo, 2016 Brasil^(31)^	Identificar los conocimientos del padre sobre la lactancia materna.	Estudio cualitativo, exploratorio y descriptivo. Recogida de datos mediante entrevistas semiestructuradas, con posterior análisis de contenido según Bardin.	N = 15, Edad = 19–51 años. Hombres con hijos de entre siete días y 11 meses. Dos habían completado estudios superiores; siete, estudios secundarios; dos no habían completado estudios secundarios y cuatro no habían completado estudios primarios. Ocho estaban en unión estable y siete estaban casados.
Brown, 2014 Reino Unido^(32)^	Explorar las experiencias de los padres a la hora de apoyar a su pareja durante la lactancia y examinar sus actitudes hacia la educación, la información y el apoyo que han recibido.	Estudio descriptivo-cualitativo simple. Cuestionario en línea compuesto por preguntas abiertas. El análisis de contenido se realizó para cada entrevista mediante lectura para identificar temas emergentes.	N = 117, edad no especificada. Padres cuyas parejas habían dado a luz en los últimos 2 años y habían iniciado la lactancia al nacer.
Canton, 2022 Argentina^(33)^	Explorar los vínculos que rodean a las madres lactantes, a partir de la valoración de la lactancia expresada por sus parejas, en una maternidad privada de la ciudad de Buenos Aires.	Estudio cualitativo basado en la teoría fundamentada. Los datos se recogieron a partir de cuatro grupos focales con padres. Los datos se procesaron mediante la definición de unidades de análisis de flujo libre, codificadas en dos niveles: codificación abierta, en categorías que surgieron y formando la agrupación de categorías en cinco temas principales.	N = 16, edad = 26 a 41 años. Padres de nivel socioeconómico medio-alto, con estudios secundarios o superiores, de nacionalidad argentina, dos de ellos extranjeros.
Fazio, 2018 Brasil^(34)^	Identificar la estructura y el contenido de las representaciones sociales de los padres sobre la alimentación y la lactancia materna exclusiva y analizar y analizar las relaciones estructurales entre estas representaciones.	Estudio descriptivo con enfoque cualitativo, basado en la Teoría de las Representaciones Sociales. Los datos se recogieron mediante evocaciones libres y entrevistas semiestructuradas. El análisis utilizó la codificación por unidades de contexto.	N = 54, edad = 18 a 50 años 23 padres habían completado/no completado la educación primaria, 24 habían completado la educación secundaria y 7 la educación superior. 51 vivían con su pareja y 3 no vivían con su pareja. Todos tenían hijos y 15 tenían al menos un hijo de relaciones anteriores.
Gutmann, 2018 Brasil^(35)^	Identificar la contribución del padre a la lactancia materna y al cuidado del recién nacido.	Estudio exploratorio, descriptivo y de enfoque cualitativo. Los datos se recogieron mediante entrevistas semiestructuradas. Se utilizó el análisis de contenido según Bardin.	N = 30, edad = 18–50. La mayoría había terminado la enseñanza secundaria y vivía con su pareja.
Lima, 2020 Brasil^(36)^	Identificar las percepciones de los padres sobre la lactancia materna.	Estudio descriptivo. Entrevistas semiestructuradas, presenciales o telefónicas, con posterior análisis de contenido según Bardin.	N = 11, edad = 21 a 26 años. Padres con estudios primarios completos, etnia blanca autodeclarada, ingresos familiares de 1 salario mínimo, que declararon acudir a las citas prenatales.
Merritt, 2019 Inglaterra^(37)^	Explorar las creencias, actitudes y comportamientos de los padres en relación con la lactancia materna y cómo influyen positiva o negativamente en la decisión de sus parejas de iniciar o continuar la lactancia.	Estudio descriptivo y cualitativo. Entrevistas individuales con los padres, que pueden estar acompañados por sus parejas. Análisis mediante el método comparativo constante.	N = 18, edad = 21 a 42 años, media 33,6 años. Todos los participantes se clasificaron como «Blancos - Británicos», excepto cuatro que se identificaron como «Blancos - Otros». La mayoría tenía un título universitario (n = 13). Sólo uno se clasificó como desempleado. Cuatro entrevistados trabajaban para las Fuerzas Armadas.
Mitchell-Box, 2012 Estados Unidos^(38)^	Explorar las percepciones de las parejas masculinas sobre la lactancia materna para informar el desarrollo de intervenciones para aumentar su apoyo a la lactancia materna.	Estudio cualitativo, basado en la teoría fundamentada. Las entrevistas semiestructuradas se analizaron mediante la técnica de Charmaz.	N = 14, edad = 20–45 años. Una muestra de conveniencia de 14 parejas masculinas de mujeres embarazadas o puérperas con bajos ingresos. Padres que esperaban un hijo o que tenían un hijo menor de 1 año.
Mithani, 2015 Paquistán^(39)^	Explorar las percepciones de los padres sobre la lactancia materna.	Estudio exploratorio con enfoque cualitativo. Se realizaron entrevistas semiestructuradas. El análisis temático se llevó a cabo utilizando las seis etapas del proceso iterativo.	N = 12, con una edad no especificada, seis participantes (padres) de la zona urbana y seis (padres) de la zona semiurbana. Además, los padres de la muestra urbana eran mayores que los de la zona semiurbana.
Pinto, 2018 Brasil^(40)^	Conocer la percepción paterna de las dificultades de la lactancia materna.	Investigación descriptiva, con enfoque cualitativo, mediante entrevistas semiestructuradas. Los datos se analizaron mediante la técnica del Discurso del Sujeto Colectivo.	N = 12, con edades comprendidas entre 30 y 28 años. Padres que tenían hijos a su cargo y cuyo binomio madre/hijo presentaba alguna dificultad en la lactancia. Todos los padres vivían con sus familias, ocho estaban casados y cuatro en unión estable. La mayoría había terminado la enseñanza secundaria y todos tenían un empleo remunerado.
Resende, 2014 Brasil^(41)^	Presentar los sentimientos mencionados por los padres y sus contribuciones al proceso de lactancia.	Estudio cualitativo y fenomenológico. Se realizaron entrevistas con preguntas orientativas. Los puntos obtenidos en ellas se analizaron junto con la lista de contribuciones comunicadas por los padres.	N = 40, Edad = 19–58 años. Padres cuyos hijos fueron amamantados entre 6 meses y 2 años. Padres cuyas parejas estaban amamantando.
Teston, 2018 Brasil^(42)^	Comprender cómo reciben los padres su papel en relación con la lactancia materna.	Estudio descriptivo y cualitativo. Los datos se recogieron mediante entrevistas con una pregunta orientadora y dos preguntas de apoyo. Los datos se sometieron a un análisis de contenido temático.	N =14, edad = 18–35, media 30 años. Siete de ellos eran parturientas, 57,1% eran del municipio donde estaba localizada la institución; 42,8% eran blancas, 35,71% morenas y 21,42% negras; 82% tenían por lo menos seis años de escolaridad.

Fuente: autoría propia.

La calidad de los estudios incluidos en la metasíntesis fue satisfactoria (Cuadro 3). Todos ellos presentaban claramente el objetivo, la metodología y los resultados, así como la estrategia de reclutamiento para alcanzar los objetivos de la investigación. La mayoría, sin embargo, no proporcionaba información sobre la relación entre investigador y participante. Sólo tres artículos describían parcialmente las cuestiones éticas utilizadas y tres describían parcialmente las contribuciones de la investigación a la práctica.

**Cuadro 3:** Resultados de la evaluación de la calidad de los artículos (n = 13) – Teresina, PI, Brasil, 2024.

(Primer autor, año)	Items									
	1	2	3	4	5	6	7	8	9	10
Aguilera-Díaz, 2023^(30)^	✓	✓	✓	✓	✓	✓	✓	✓	✓	✓
Azevedo, 2016^(31)^	✓	✓	✓	✓	✓	X	✓	✓	✓	X
Brown, 2014^(32)^	✓	✓	✓	✓	✓	✓	X	X	✓	✓
Canton, 2022^(33)^	✓	✓	✓	✓	✓	✓	✓	✓	✓	✓
Fazio, 2018^(34)^	✓	✓	✓	✓	✓	X	✓	✓	✓	✓
Gutmann, 2018^(35)^	✓	✓	✓	✓	✓	X	✓	X	✓	✓
Lima, 2020^(36)^	✓	✓	✓	✓	✓	X	✓	✓	✓	✓
Merritt, 2019^(37)^	✓	✓	✓	✓	✓	X	X	✓	✓	X
Mitchell-Box, 2012^(38)^	✓	✓	✓	✓	✓	✓	✓	✓	✓	X
Mithani, 2015^(39)^	✓	✓	✓	✓	X	X	X	X	✓	✓
Pinto, 2018^(40)^	✓	✓	✓	✓	✓	X	✓	✓	✓	✓
Resende, 2014^(41)^	✓	✓	✓	✓	✓	X	✓	✓	✓	✓
Teston, 2018^(42)^	✓	✓	✓	✓	✓	✓	✓	✓	✓	✓

Fuente: autoría propia.

Nota: ✓ – Sí; X – Informado parcialmente o No.

1. ¿Se informó claramente de los objetivos de la investigación?

2. ¿Fue adecuada la metodología cualitativa?

3. ¿Fue adecuado el diseño de la investigación para alcanzar los objetivos propuestos?

4. ¿Fue adecuada la estrategia de reclutamiento para los objetivos de la investigación?

5. ¿Se recogieron los datos de forma que respondieran a la pregunta de investigación?

6. ¿Se tuvo debidamente en cuenta la relación entre el investigador y los participantes?

7. ¿Se tuvieron en cuenta las cuestiones éticas?

8. ¿Fue suficientemente riguroso el análisis de los datos?

9. ¿Se comunicaron claramente los resultados?

10. ¿Aporta algo la investigación?

### Síntesis Temática

El análisis de los 13 artículos condujo a la creación de dos temas analíticos, que describen la influencia paterna en la decisión de mantener o interrumpir la lactancia: «Dificultad para participar activamente en el proceso de lactancia» e «Interacción directa o indirecta con la madre en la lactancia» (Cuadro 4).

**Cuadro 4:** Temas analíticos y descriptivos que califican la influencia paterna en la decisión de mantener o interrumpir la lactancia materna – Teresina, PI, Brasil, 2024.

Temas analíticos	Temas descriptivos
Dificultad para participar activamente en el proceso de lactancia.	– Un papel de apoyo en la decisión de amamantar; – Impotencia y sufrimiento ante las dificultades de la lactancia materna; – (Des)conocimiento de los beneficios de la lactancia materna; – Sentirse excluido del proceso de lactancia.
Actuar directa o indirectamente con la madre en la lactancia	– Fomento de la lactancia materna; – Apoyo al uso de sucedáneos de la leche materna; – Vergüenza cuando su pareja amamanta en público.

Fuente: autoría propia.

### Dificultad para implicarse activamente en el proceso de lactancia

En el primer tema analítico, los resultados describen el papel desempeñado por los padres en la decisión de continuar o interrumpir la lactancia, permitiendo identificar sus conocimientos sobre la lactancia y sus sentimientos de impotencia, sufrimiento y exclusión en este proceso.

#### Papel de apoyo en la decisión de amamantar

Los estudios han demostrado que los padres desempeñan un papel de apoyo en el proceso de decisión de mantener o interrumpir la lactancia materna, actuando en un papel secundario en el que a veces se abstienen de esta decisión. Algunos estudios han mostrado un apoyo incondicional a la pareja en este sentido, considerando que la madre conoce mejor sus límites y sabe qué decisión tomar^(37,38)^. A veces, el padre se abstiene de ayudarla a tomar esta decisión, ya que ve su papel como secundario en el proceso de lactancia y considera que es una decisión exclusiva de la madre^(37,38)^. Otros estudios han demostrado que algunas parejas no discuten este tema, también porque creen que la madre sabe lo que es mejor, ya que es su cuerpo^(30)^. «Ella lidia con el dolor de amamantar y pasa mucho tiempo amamantando, lo que se torna cansador para la madre: es la esposa, la mujer, la que tiene que lidiar con el dolor de amamantar, cómo duele, y la mujer la que realmente tiene que decidir»^(38)^.

Por otro lado, los padres esperan que las madres den el pecho porque creen que es la mejor forma de alimentar a su hijo, y esta percepción se basa en la información adquirida durante el embarazo^(32)^. Algunos estudios han informado de situaciones en las que la madre no quería dar el pecho y el hombre la cuestionaba por esta decisión^(38)^. De este modo, la preocupación por la nutrición del niño también lleva al padre a vigilar la lactancia^(32)^. «Ella se pone nerviosa porque lo pone al pecho, luego su boca sigue dormida, dice que está mamando, pero su boca no se mueve? Entonces le digo: ¡no lo creo! Entonces ella me dijo que me alejara. Quiero estar segura, ¿no se muere de hambre? Porque todavía no puede hablar»^(42)^.

#### Impotencia y sufrimiento ante las dificultades de la lactancia materna

Ante las dificultades de la madre en el proceso de lactancia, los padres manifestaron preocupación, tristeza, nerviosismo y un sentimiento de impotencia y frustración^(40)^. Dijeron que no sabían cómo ayudarlas^(37,40)^. Como resultado, expresaron sentimientos negativos hacia la pareja durante el periodo de lactancia y se preocuparon por no saber más información sobre el tema^(30)^. Intentaron animarlas a no rendirse y sufrieron juntas ante los problemas o dificultades relacionados con la lactancia, como el dolor cuando el bebé se prende^(40)^. También expusieron las dificultades a las que se enfrentaban las mujeres durante el proceso de adaptación a la lactancia^(38)^. Informaron de que era difícil ver a sus parejas luchando por dar el pecho y no poder hacer nada al respecto, sintiéndose culpables cuando su pareja sentía dolor, como cuando el bebé mordía el pecho de la madre^(32)^. Algunas declararon haber comprado una tetina intermedia para ayudar al bebé a mamar, evitando que la madre sintiera dolor^(40)^. El dolor que sentía la madre era lo que ayudaba a los padres a comprender que la lactancia no era fácil^(40,42)^. Además del llanto del bebé, la madre siente dolor en el estómago y las posturas para amamantar no son cómodas^(42)^. Los padres querían ayudar en este proceso, ocupándose de las tareas domésticas o buscando información sobre lo que debían hacer para ayudar a sus esposas^(35)^. Sin embargo, algunos se sentían molestos e impotentes porque querían ayudar en el proceso de lactancia, pero no sabían cómo. Según ellas, a veces los profesionales no validaban los sentimientos de los padres^(32)^. «Cuando le dije a una enfermera que me sentía impotente, me dijo que disfrutara del descanso y se rió, como si no tuviera nada que ver conmigo»^(32)^. En general, los hombres creen que las parejas no están preparadas para afrontar juntas las dificultades de la lactancia y consideran que la mejor estrategia es prepararse esperando la peor experiencia^(37)^. Afirman que esta experiencia es diferente con cada hijo^(35)^. También señalaron que, ante las dificultades encontradas, sus parejas a veces mostraban signos de agotamiento, lloraban y se ponían nerviosas^(31,32,42)^. En ese momento, solicitaron orientación a los profesionales sanitarios sobre cómo agarrarse correctamente al pecho para evitar el dolor durante la lactancia^(31,32,42)^.

#### (DES)conocimiento de los beneficios de la lactancia materna

La mayoría afirmó que el mejor alimento para su hijo es la leche materna y declararon saberlo incluso antes de acudir a las consultas prenatales^(37)^. En los estudios analizados, eran conscientes de que la leche materna cambia su composición a lo largo de la lactancia, aportando diferentes nutrientes y componentes adecuados para cada etapa del desarrollo del niño^(32,36)^. «Es un alimento completo y tiene todos los nutrientes necesarios que el bebé necesita. ¡Es el mejor alimento que existe! Más aún para que se ponga fuerte, para que adquiera resistencia, ¿no?»^(40)^. También declararon creer que la leche materna es la mejor leche porque ayuda al niño a crecer más fuerte, previene enfermedades y ayuda a los padres a ahorrar dinero^(31,36,39)^. En consecuencia, creen que debe ser el alimento principal del niño hasta los 6 meses^(31)^, y que el bebé debe seguir siendo amamantado hasta los dos años^(31,41)^. Sin embargo, algunas mencionaron que no comprendían los beneficios de la lactancia materna y que sólo sabían que era importante para la salud del bebé^(31,36)^. Afirmaron no haber recibido ninguna orientación durante la atención prenatal. «Nadie habló de la lactancia materna durante la atención prenatal»; “No recuerdo que me dieran ninguna orientación sobre la lactancia materna”^(42)^. «No tenía ninguna información y ni siquiera busqué más información»^(40)^. También hubo discrepancias sobre las orientaciones dadas a los padres por los distintos profesionales sanitarios. En este sentido, les gustaría que los profesionales proporcionaran la misma orientación, ya que se sienten confusos en medio del torbellino de directrices diferentes^(37,42)^. Otros afirmaron sentirse excluidos, a pesar de participar en los cuidados prenatales: «Toda la información iba dirigida a mi mujer. Lo que podía comer, hacer, probar, etc. Sé que ella era la protagonista, pero yo sentía que no tenía nada que ver conmigo»^(32)^.

#### Sentirse excluido del proceso de lactancia

Algunos padres dijeron que echaban de menos tener el mismo vínculo con su bebé que la madre, porque creían que la mujer ya tiene un vínculo natural con su hijo^(37)^. «Porque la madre ya tiene un vínculo natural. Lo que es el embarazo, nueve meses con ella, todo se desarrolla ahí con ella, ¿no? El padre es sólo un espectador. Él mira...»^(31)^.

Aunque algunas encontraban hermoso el vínculo entre madre e hijo, se sentían excluidas de la lactancia^(32,37)^. «Sé que no debería haberme sentido así, pero hasta que mi mujer empezó con la lactancia mixta yo... me sentía, y sé de nuevo que no debería haberme sentido así, pero me sentía apartado de la situación»^(37)^.

### Actuación directa o indirecta con la madre durante la lactancia

El segundo tema analítico describe las acciones participativas del padre para fomentar la lactancia materna o los sucedáneos de la leche materna y su incomodidad con la lactancia materna en público.

#### Fomento de la lactancia materna

Los hombres fomentan el proceso de lactancia proporcionando apoyo al binomio madre-hijo^(36)^. Este estímulo puede realizarse a través de gestos, como hacer que ambos se sientan cómodos y proporcionar un entorno tranquilo y silencioso para que la madre pueda amamantar cómodamente. Estas actitudes paternas convierten al padre en parte fundamental del proceso de lactancia, asumiendo el papel de apoyo para que la madre no desista y se mantenga firme en este empeño^(35,40)^. Para algunos, el papel paterno en la lactancia requiere paciencia, ya que a menudo se sienten muy cansados y aun así tienen que coger al bebé, llevarlo a amamantar y luego ponerlo en la cuna varias veces por noche^(31)^. En los estudios analizados, algunos padres afirmaron que se consideraban importantes en el proceso de lactancia^(30)^. «Sí, yo era importante [...] No físicamente, pero sí en términos de supervisión, etc.»^(30)^. Algunas relataron no estar presentes porque trabajan mucho y eso dificulta su participación^(31)^. Con toda esta demanda paterna durante la lactancia, los padres creen que el permiso de paternidad podría facilitar su participación en la lactancia, ya que podrían cuidar de los otros hijos y realizar algunas tareas domésticas, además de acompañar a la mujer en todo momento^(39,41)^. Estos padres también creían que podían ayudar buscando instrucciones sobre los principales aspectos del cuidado del bebé y de la madre, así como proporcionándose estrategias para transformar el hogar en un entorno tranquilo y acogedor^(41)^. Una de las participantes señaló que estaba al lado de la madre y abrazaba al bebé mientras lo amamantaba^(30)^. «Normalmente también me acercaba a él y le tocaba la frente o la mejilla, para poder participar también en el acto»^(30)^. Algunas creían que luchar contra la violencia doméstica que habían ejercido previamente contra sus parejas antes del nacimiento de sus hijos era también una estrategia para apoyar la lactancia^(39)^. «Siempre apoyo a mi mujer en la lactancia... Nunca le hago daño. Incluso cuando quiero pegarle, no lo hago porque sé que es muy frágil»^(39)^. Por otro lado, algunos padres se sentían incapaces de desempeñar ningún papel directo en la lactancia, ya que consideraban que era responsabilidad exclusiva de la madre promoverla. Para ellos, el papel que asumían era simplemente de apoyo a la familia, porque así la madre no tendría que trabajar y estaría a disposición del bebé en cualquier momento^(31,39)^. También declararon que querían que sus esposas se quedaran en casa para mantener el proceso de lactancia^(33)^. «Sería estupendo que ella se quedara en casa y yo trabajara..., pero los roles tradicionales de género no se pueden conseguir económicamente»^(33)^.

#### Apoyo al uso de sucedáneos de la leche materna

Algunos padres creían que la leche materna no era suficiente para alimentar a sus hijos^(34)^y que debían utilizar leche artificial, ya que era suficiente para las necesidades nutricionales del bebé^(32)^. Según ellos, el uso de sucedáneos de la leche materna era más sano y cómodo, y el niño ganaba más peso^(39)^. Algunas incluso dijeron que no veían ninguna diferencia entre la leche materna y la de fórmula, ya que pensaban que ambas tenían los mismos beneficios para nutrir a sus hijos y, por lo tanto, optaron por utilizar leche de fórmula^(32)^. En algunos casos, se describió que el uso de leche artificial aportaba varios beneficios, como la practicidad a la hora de alimentar al niño, la libertad de salir más a menudo de casa con la pareja y la percepción de que contribuía a que el bebé durmiera más tiempo^(38)^. Los padres también querían participar más en la lactancia y, por lo tanto, afirmaron que se sentían más unidos a sus hijos cuando los alimentaban con biberón^(32,37)^. Otro beneficio que citaron fue el de turnarse para alimentar al bebé, ya que el uso de sucedáneos de la leche les permitía ayudar a sus parejas en esta tarea, lo que no podía hacerse con la lactancia materna exclusiva^(38)^. Algunas, para sentirse más incluidas en el proceso de alimentación de sus hijos, optaron por introducir el biberón, ya que querían sentirse útiles e incluidas en el proceso de lactancia^(32,35)^. Con ello, sentían que estaban dando a sus hijos algún tipo de placer y cuidado, más allá de acciones como cambiar pañales y bañarlos^(37)^. Además, afirmaron darse cuenta de la importancia de la presencia paterna durante el proceso de lactancia y a lo largo del crecimiento de sus hijos^(30,37)^. Según ellas, como echaban de menos a sus padres cuando eran jóvenes, querían estar más presentes en la vida de sus hijos^(33)^. Otros estudios, en cambio, describieron que los padres entendían que la leche de fórmula no era saludable para sus hijos, ya que podía causar diarrea y desnutrición^(39)^. En este sentido, los padres se refirieron a este alimento como un producto artificial, capaz de causar daño al niño, además de ser una fuente de soja^(30)^. Otra desventaja citada fue el elevado coste, ya que los bebés toman mucha leche en los primeros seis meses de vida^(32, 39-41)^. Por ello, muchos prefieren que sus esposas sigan amamantando, ya que esto les ayuda a ahorrar dinero^(32,40)^.

#### Incomodidad cuando la pareja da el pecho en público

Algunos padres se sentían incómodos al ver a sus parejas amamantando delante de amigos y familiares, aunque fuera algo natural, porque asociaban la lactancia con algo sexual o pervertido^(32,38)^. Algunos pedían a sus parejas que intentaran esconder el pecho: «Inténtalo, escóndelo todo lo que puedas, supongo»^(38)^. Muchos preferían que la mujer diera el pecho en presencia de extraños y no de conocidos, ya que creían que la vergüenza sería mayor para los que miraban^(32,38)^. A otras no les gustaba que diera el pecho delante de sus hijos mayores, pues creían que ya no tenían edad para ver los pechos de su madre^(38)^.

## DISCUSIÓN

Esta revisión permitió sintetizar la evidencia científica cualitativa sobre la influencia del padre en la decisión de continuar o interrumpir la lactancia materna. Se establecieron dos temas analíticos, destacando el conocimiento de los padres sobre la lactancia materna, su influencia en la decisión de continuar o interrumpir la lactancia y los sentimientos y dificultades que experimentan durante la lactancia. Esta metasíntesis también identificó cómo se sienten los padres al amamantar en público y qué factores interconectados interfieren en este proceso, influenciados por la masculinidad, que también se ve afectada a lo largo del proceso.

Los resultados corroboran otras revisiones, en las que la implicación de la pareja durante la lactancia puede contribuir a mantener o interrumpir este proceso, además de ampliar el abordaje de las dificultades a las que se enfrentan los padres durante este periodo, como la falta de información y el miedo^(43)^. La implicación del padre puede favorecer la lactancia, ya que puede ayudar en las tareas domésticas y en el cuidado de otros niños, para que la madre pueda amamantar. Además, cuando tiene acceso a información sobre la lactancia, puede animar a su mujer y favorecer así la continuación de la lactancia^(18)^. Incluso el apoyo verbal de la pareja se asocia a una mayor duración y exclusividad de la lactancia^(19)^. El reconocimiento por parte de los padres de que la leche materna es el alimento más eficaz y nutritivo para el desarrollo del bebé también les ayuda a animar a sus parejas a continuar con la lactancia, a pesar de las posibles dificultades que puedan encontrar^(44)^. La experiencia paterna de la lactancia, sin embargo, también está impregnada de diversos sentimientos ambiguos, ya que se sienten felices y quieren apoyar la lactancia, mientras que al mismo tiempo se sienten frustrados y excluidos^(45)^. Esto se debe, entre otras razones, al hecho de que la decisión final sobre si amamantar o no recae exclusivamente en la madre, y los padres se sienten excluidos porque no saben cómo participar en este proceso^(14,17,45,46)^. La investigación ha demostrado que los padres sienten algunas limitaciones a la hora de apoyar a sus parejas en el proceso de lactancia, incluida la presencia durante la misma, debido a las construcciones sociales relacionadas con la masculinidad^(47)^. Los estudios informan de que se presta poca o ninguna atención a los padres durante la atención prenatal y, como resultado, acaban por no recibir suficiente información sobre la lactancia, así como algunos no acuden a las citas de forma regular^(45)^. La investigación también muestra la necesidad de más estrategias educativas para implicar a los padres durante el periodo de lactancia^(48,49)^. Involucrar a los padres en la atención prenatal es de suma importancia, ya que en este momento reciben información sobre los beneficios de la leche materna y pueden ayudar y animar a sus parejas durante el proceso de lactancia^(18)^. Este seguimiento puede aliviar el impacto psicológico y emocional, ya que comienzan a asumir nuevos roles familiares, de padre y madre, y el acceso a la información permite darse cuenta de que pueden desempeñar un papel importante en la lactancia, reforzando el vínculo familiar padre-madre-hijo^(50)^. Por otro lado, se necesitan estrategias para llegar a los padres que afirman no tener tiempo para el seguimiento, o que no muestran interés en hacerlo, porque el acceso a la información puede afectar directamente a su opinión sobre la lactancia materna^(11,18)^.

Con la llegada de un hijo, la rutina familiar cambia, incluida la vida sexual de la pareja, ya que la madre tiene que prestar más atención al recién nacido, por lo que muchos padres se sienten excluidos de la rutina diaria. Para hacer frente a esta situación, algunos padres ayudan en la lactancia, mientras que otros prefieren mantenerse al margen de este proceso, alegando que no son ellos los que amamantan^(44)^. Esta es la primera meta-síntesis cualitativa que revisa sistemáticamente la literatura sobre la influencia paterna en el proceso de toma de decisiones sobre la lactancia materna. El rigor empleado en el método de esta revisión, que utilizó la herramienta SPIDER, y la inclusión de cuatro bases de datos complementadas con búsquedas manuales son puntos fuertes de esta investigación. Desde la última década, se ha incrementado el fomento de la participación paterna en las actividades de cuidado, incluyendo el apoyo a la lactancia materna^(4)^. Sin embargo, el número de estudios en la muestra final, que refleja las tendencias recientes sobre el tema, puede ser una limitación de esta revisión, que abre perspectivas para futuros estudios en países de ingresos altos, medios y bajos, y subraya la necesidad de más investigación centrada en la influencia paterna en el proceso de toma de decisiones sobre la lactancia y en la participación de los padres durante la lactancia en su conjunto.

En general, los padres reciben poca información sobre la lactancia y se sienten excluidos o no saben cómo participar en este proceso. En este contexto, esta meta-síntesis ayuda a los profesionales de la salud, especialmente a las enfermeras, a involucrar a los padres desde la atención prenatal para identificar las necesidades informativas y emocionales relacionadas con la lactancia materna, con el fin de establecer intervenciones de apoyo a favor de la autoeficacia e inclusión de los padres, así como su papel en la toma de decisiones y el estímulo para mantener la lactancia materna. Esto ayudará a aliviar los miedos y la ansiedad de los padres, además de contribuir a la continuación de la lactancia materna exclusiva.

## CONCLUSIÓN

Esta revisión ha analizado y sintetizado el conocimiento publicado en los últimos 10 años sobre la influencia de los padres en la decisión de mantener o abandonar la lactancia materna de sus hijos. Se trata de una experiencia compleja, en la que el hombre asume un nuevo rol, el de ser padre, y esto genera miedo e inquietud. La decisión de dejar o continuar la lactancia la suele tomar la madre, y el padre la apoya en esta decisión. El padre también se siente excluido de este proceso, así como confuso sobre lo que debe hacer para ayudar a su pareja y a su hijo.

El padre fue representado como muy importante durante la lactancia y puede ayudar a lo largo de este proceso cuando tiene acceso a la información, especialmente durante la atención prenatal, ya que esta orientación alivia el dilema de saber cómo participar y ser incluido durante la lactancia. Aunque ven la lactancia como una experiencia dolorosa y difícil para sus parejas, consideran que la leche materna es el mejor alimento para sus hijos. Además, aunque algunos de ellos ayudan con las tareas domésticas y otros asuntos para facilitar el proceso de lactancia a sus parejas, otros no saben cómo participar en el proceso de lactancia y se sienten excluidos. En este sentido, los padres pueden y deben ser incluidos en el proceso de lactancia, y los profesionales sanitarios deben incluirlos desde la atención prenatal para favorecer el vínculo entre marido y mujer, así como la continuidad de la lactancia.
